# Integrative proteome and metabolome analyses reveal molecular basis of the tail resorption during the metamorphic climax of *Nanorana pleskei*

**DOI:** 10.3389/fcell.2024.1431173

**Published:** 2024-08-19

**Authors:** Tao Zhang, Lun Jia, Xinying Li, Zhiyi Niu, Siping Zhang, Weijun Dong, Liang Peng, Miaojun Ma, Huihui Wang, Xiaolong Tang, Qiang Chen

**Affiliations:** ^1^ Department of Animal and Biomedical Sciences, School of Life Sciences, Lanzhou University, Lanzhou, China; ^2^ Ministry of Education Key Laboratory of Cell Activities and Stress Adaptations, School of Life Sciences, Lanzhou University, Lanzhou, China; ^3^ Gansu Key Laboratory of Gene Editing for Breeding, School of Life Sciences, Lanzhou University, Lanzhou, China; ^4^ State Key Laboratory of Herbage Improvement and Grassland Agro-ecosystems, College of Ecology, Lanzhou University, Lanzhou, China; ^5^ School of Stomatology, Lanzhou University, Lanzhou, China

**Keywords:** amphibian, Qinghai-Tibet plateau, integrative omics, proteomic, metabolomic, tail resorption, metamorphosis, different portions

## Abstract

During the metamorphosis of anuran amphibians, the tail resorption process is a necessary and crucial change. One subject that has received relatively little or no attention is the expression patterns of proteins and metabolites in the different tail portions during metamorphosis, especially in highland amphibians. The mechanisms of tail resorption in three portions (the tip, middle and root) of the tail were investigated in *N. pleskei* G43 tadpole based on two omics (proteomic and metabolomic). Integrin αVβ3 was found to be high expressed in the distal portion of the tail, which could improve the sensitiveness to thyroid hormones in the distal portion of the tail. Muscle regression displayed a spatial pattern with stronger regression in distal and weaker one in proximal portion. Probably, this stronger regression was mainly performed by the proteases of proteasome from the active translation by ribosomes. The suicide model and murder model coexisted in the tail resorption. Meanwhile, fatty acids, amino acids, pyrimidine, and purine which derived from the breakdown of tissues can be used as building blocks or energy source for successful metamorphosis. Our data improved a better comprehension of the tail resorption mechanisms underlying the metamorphism of *N. pleskei* tadpole through identifying important participating proteins and metabolites.

## 1 Introduction

Tissues and organs of anuran amphibians must undergo extensive changes during the metamorphosis, including the tail resorption process, which aids in adaptation to the terrestrial environments. The metamorphosis can serve as a good model for the investigation of development and apoptosis since it is characterized by drastic transformation of juvenile tissues and organs into adult ones (Liu et al., 2016).

Three models have been proposed to explain the phenomenon of tail resorption in anuran tadpoles: the immunological rejection model (Nakai et al., 2016), the murder model and the suicide model (Nakai et al., 2017). Extracellular matrix (ECM) degradation is driven by the matrix degrading proteinases which are released by subepidermal fibroblasts. This detaches muscle cells from the matrix and results in cell death (Berry et al., 1998; Nakajima et al., 2005). This is defined as murder model. In contrast, the suicide model posits that the death of tadpole muscle cells occurs through conserved programmed mechanisms that are triggered by the overexpression of genes related to cell death. Both the murder and suicide model, which can be utilized separately or in combination, are dependent on thyroid hormone (TH). A study has demonstrated that physiological TH concentrations caused apoptosis in a myoblastic cell line from *Xenopus laevis* tadpole tail. As the cell line lacked subepidermal fibroblasts, this result provided further evidence for the TH-dependent suicide model (Yaoita and Nakajima, 1997; Elinson et al., 1999).

The regulation of anuran larval development and the beginning of metamorphosis were both recognized to be significantly influenced by thyroid hormones (THs) (Tata, 2006). When the tail is dramatically resorbed, THs levels peaked with a quick metamorphic transition in *X*. *laevis* tadpole (Nakajima et al., 2005). The mRNA quantities of TH Receptor β (TRβ), collagenase 3 (MMP13), stromelysin-3 (MMP11), and fibroblast activation protein α (FAPα) increased in the tail when *Xenopus* tadpole undergoes metamorphosis (Yaoita, 2019).

Proteomics is used in a variety of research contexts, and it involves analyzing, identifying, and quantifying the total amount of proteins present in cell, tissue, or organism (Aslam et al., 2017). The proteome of the tail fin of *Rana catesbeiana* tadpole revealed the differential expression of 15 proteins involved in apoptosis, extracellular matrix structure, immune system, metabolism, mechanical function, and oxygen transport during the process of T3-induced (48 h exposure) tail resorption (Domanski and Helbing, 2007).

When responding to endocrine signals and environmental variations, metabolism is the final stage of cellular regulatory cascades (Bundy et al., 2009). Adequate biosynthesis is required for organogenesis and organ remodeling (Mukhi et al., 2010; Tennessen et al., 2011; Ryall, 2013; Cliff and Dalton, 2017; Krejci and Tennessen, 2017). Carbohydrates, lipids, and amino acids, the three main types of metabolic substrates, can all be utilized as fuel to produce energy, but their functions in metabolite interconversion and biosynthesis appear to differ (Vander Heiden et al., 2009). Fat serves as the main source of energy during famine in pro-metamorphic tadpoles (Wright et al., 2011; Zhu et al., 2019). The tail and gills of tadpoles that are actively degenerated during metamorphic climax can be used as additional energy storage (Hourdry et al., 1996). Comparative metabolomics of *Rana omeimontis* tadpoles revealed that the entire tail had maintained glycogenolysis, stimulated metabolic activity (fatty acid elongation and desaturation, the synthesis of bioactive metabolites), and repressed biological processes (protein synthesis, glycolysis, β-oxidation, transamination, energy production and consumption) during the metamorphic climax (Zhu et al., 2020).

Many studies have employed a combination of metabolomics and proteomics to gain full insight into biological events. It is crucial to ascertain whether proteins and metabolites play a role in controlling the processes of tail resorption and whether the altered expression of some proteins and metabolites exist between the different sections of the tail. Overall, many researches have been conducted on the processes of the degeneration of tail tissues, but none of these have been done on the indigenous species of the Qinghai-Tibet Plateau. Importantly, the mechanisms behind the tail resorption of different portions in anuran amphibians have remained vacant.

As one of the major predators *N. pleskei* is very important to the local ecosystem. The majority of the completed researches on this species concentrates on taxonomy, habitat, population dynamics, molecular evolution, cold hardiness and response to heat wave (Dai et al., 2005; Wang B. et al., 2017a; Wang G. et al., 2017b; Zhou et al., 2017; Niu et al., 2018; Wang et al., 2018; He et al., 2021; Zhang et al., 2021; Zhang et al., 2023). Investigations on the process and mechanism of the degeneration of tail tissues over metamorphosis have not been reported. In the swampy regions of the Qinghai-Tibetan Plateau at altitudes of 3,300 to 4,500 m above sea level, *N*. *pleskei* lives in puddles, streams, and their surroundings (Fei et al., 2012; Che et al., 2020). The spawning season lasts from mid-May to early-June in this species. Breeding female frogs lay eggs in puddles, ponds, and other static waterways. The tadpoles are primarily benthic and inhabit still water ponds (Che and Wang, 2016). Metamorphosis occurs at Gosner stages 41–46 (G41-G46) (Gosner, 1960). During this period the tail gradually loses its function of providing propulsion for swimming and is resorbed as a source of energy and building materials (Yaoita, 2019).

A large amount of data is available on the mechanisms of the tail resorption mechanisms in *X. laevis* and *Xenopus tropicalis*. However, the living environments of these *Xenopus* is very different from that of *Nanorana pleski*. Do they employ similar strategies to resorb the tail? Is there any common character or different character in their tail resorption? We try to explore it in this study. Our field observations showed that tail resorption of *N. pleskei* tadpole was starting at G43 stage and that the tail length was rapidly decreased to nearly nothing in about 2 days. The present study compares the differentially accumulated metabolites (DAMs) and differentially expressed proteins (DEPs) between three portions (tip, middle and root) of the tail of *N*. *pleskei* G43 tadpoles during metamorphosis. In parallel, additional experimental omics data can be accumulated to deepen our understanding of anuran amphibian development.

## 2 Material and methods

### 2.1 Tissue collection

**FIGURE 1:**
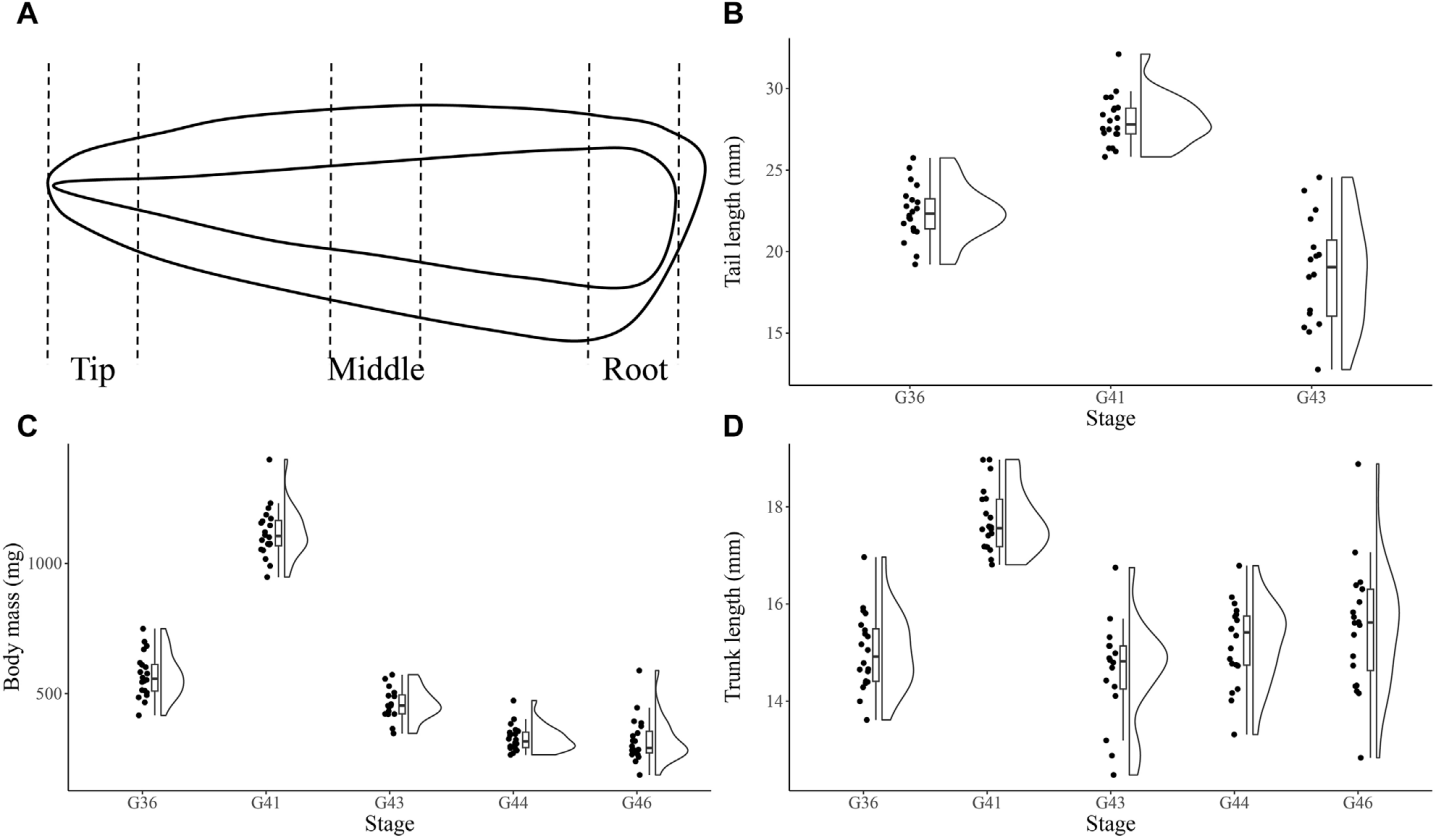
The sampling schematic and growth index of *Nanorana pleskei* tadpoles. The sketch of collected the tip, middle and root portions from the whole G43 tail of the tadpoles **(A)**. Tail length of G36, G41 and G43 tadpoles **(B)**. Body mass of G36, G41, G43, G44 and G46 tadpoles **(C)**. Trunk length of G36, G41 and G43 tadpoles **(D)**.

Tadpoles (G36, G41, G43, G44 and G46 stage) of *N*. *pleskei* were collected from field in Awancang (33.795 °N, 101.76 °E, 3,501 m asl), Maqu County, Gansu Province, China, in June to August 2021. The morphological changes of the collected tadpoles was observed. We found that at G43 stage, there were significant changes and a fast resorption of the tail. Larvae were transported to laboratory at Gannan Grassland Ecosystem National Observation and Research Station near (15 km) collecting site for immediate sampling. Tadpoles of G43 stage were sacrificed to sample tails (blood was removed). The ratio of tail to trunk at G36, G41 and G43 was 1.49 ± 0.02 (n = 20), 1.58 ± 0.03 (n = 20) and 1.30 ± 0.07 (n = 16), respectively (Figures 1B, C, D). Tails were absent inG44 and G46 tadpoles. The whole G43 tails were cut into tip (T), middle (M) and root (R) portions (Figure 1A) which were then immediately frozen with liquid nitrogen for proteomic (n = 6) and metabolomic (n = 10) analysis.

### 2.2 Proteomics assay analysis

#### 2.2.1 Sample preparation

Tissues were homogenized by bowl focused ultrasonicators (longlight, Shenzhen) in lysis buffer (6M GdmCl, 10 mM TCEP, 40 mM CAA, 100 mM Tris pH8.5) in 4°C (Rappsilber et al., 2007; Kulak et al., 2014). The samples were digested by trypsin (YAXINBIO, Shanghai) with radio of 1:50 for 18 h at 37°C. The samples were dried at 65°C in a rotary vacuum evaporator and stored at −80°C. When used, the samples were resuspended in 30 μL 0.1% formic acid (FA). Protein concentration was obtained by bicinchoninic acid (BCA) assay.

#### 2.2.2 Mass spectrometry assay

Mass spectrometry measurements were performed by an Orbitrap Fusion Lumos mass spectrometer (Thermo Fisher Scientific) connected to an EASY-nLC 1200 system with data-independent positive ion mode and 1 μg protein was injected. The mobile phases for LC include buffer A (0.1% FA) and buffer B (80% acetonitrile, 0.1% FA). The peptides were separated using a 160-min nonlinear gradient consisting of 4% buffer B for 10 min, 4%–10% buffer B for 60 min, 10%–28% buffer B for 65 min, 28%–60% buffer B for 5 min, 60%–100% buffer B for 5 min and 100% buffer B for 15 min at a flow rate of 300 nL/min.

#### 2.2.3 Statistical analyses

The DIA-NN (version 1.8.1) was used for label-free quantification data analysis (Demichev et al., 2020). MS/MS spectra were searched against protein file of *Nanorana parkeri* genome (KIZ, 2021; Fu et al., 2022). The methionine oxidation and cysteine carbamidomethylation were included as variable modification and fixed modification, respectively. Other parameters were set up using default values, and the false discovery rate was set to 0.01 for both peptide and protein identifications. The MaxLFQ algorithm (Cox et al., 2014) for label-free data-independent acquisition (DIA) mass spectrometry-based proteomics was implemented in iq R package (version 1.9.3) (Pham et al., 2020) using default settings to estimate protein quantification. The proteomics statistical and data analyses were performed using protti (version 0.3.0) (Quast et al., 2021) with a moderated *t*-test based on limma R package to find differentially expressed protein (DEP). DEP was defined as adjusted *p*-value <0.05 and absolute value of log_2_(fold change) > 1.

### 2.3 Metabolomic assay analysis

#### 2.3.1 Sample preparation

The samples were homogenized in liquid nitrogen using JXFSTPRP Tissuelyser (Jingxin, Shanghai, China). Prechilled methanol: acetonitrile: water (2:2:1, v/v) with radio of 1:50 (w/v) was added to tissue powder followed by ultrasonication for 30 min × 2 at 4°C (SCIENTZ08-III non-contact type ultrasonic cell crusher, Scientz, Shanghai, China) and incubation at −20°C for 1 h. After centrifugation at 12,000 rpm for 15 min (4°C), 200 μL supernatant was transferred into a new tube and freeze-dried. 20 μL of each sample was transferred into new tube as quality control (QC) sample and freeze-dried. Multiple uniformly spaced insertions of QC samples into experimental samples were utilized when sequencing, and data of QC samples were used to evaluate the stability of instrument. Samples were reconstituted in 200 μL ice-cold acetonitrile: water (1:1, v/v), and centrifuged for 25 min at 15,000 rpm at 4°C to remove insoluble debris (Want et al., 2013; Zhu et al., 2019; Mei et al., 2021).

#### 2.3.2 LC-MS-based untargeted metabolomics analysis

The supernatant fraction from sample preparation step was analyzed using liquid chromatograpy-tandem mass spectrometry (LC-MS/MS) on a Thermo Scientific Dionex UltiMate 3,000 ultrahigh pressure liquid chromatography system (ThermoFisher, USA) equipped with a Thermo Orbitrap Fusion Lumos mass spectrometer detector. The column used for the separation was a Thermo Scientific™ Hypersil GOLD™ C18 (2.1 mm, 1.9 um; Thermo Scientific, USA). The oven temperature was set to 35°C. The gradient elution involved a mobile phase consisting of 0.1% formic acid (A) in water and acetonitrile (B). The initial condition was set at 2% B and held 5 min. A linear gradient for 45 min was applied with the final of 100% B, held 5 min and followed by 5 min gradient to 2% B. Flow rate was set to 0.3 mL/min, and 5 μL of samples was injected. Mass spectra were acquired in positive ion mode and negative ion mode. In negative ion mode, solvent B mentioned above replaced by 5 mM ammonium acetate, remainder unchanged (Ivanisevic et al., 2013; Li et al., 2021).

#### 2.3.3 Mass spectrometry raw data processing

Xcalibur (version 4.0, Thermo Fisher Scientific, USA) was used to collect metabolomic data as raw format for subsequent analysis. After data acquisition, raw data of DDA (data-dependent acquisition) was converted from the vendor-specific file format (raw) into Analysis Base File format (abf) using freely available Reifycs ABF converter. After conversion, the MS-DIAL software (version 4.9.221218 windowsx64) was used for feature detection, spectral deconvolution, peak identification, and alignment between samples (Tsugawa et al., 2015). The parameters used in MS-DIAL were included in Supplementary Text S1.

#### 2.3.4 Statistical analyses

The R package muma (version 1.4) was used to get Benjamini-Hochberg-adjusted *p*-values and fold changes (FC) between groups (Gaude et al., 2013). SIMCA-14.1 (Umetrics, Kinnelon, United States) was used to perform orthogonal partial least squares discriminant analysis (OPLS-DA) for obtaining variable influence on projection (VIP) values. DAMs were defined using the cutoff criteria from previous research (Qi et al., 2022). Using the following cutoff criteria, DAM was defined: (1) VIP >1; (2) the absolute value of log2(fold change) > 1; (3) Benjamini-Hochberg-adjusted *p*-values <0.05.The Chemical Similarity Enrichment Analysis (ChemRICH) was conducted to classify metabolites into biochemical clusters from metabolomic database using Chemical Translation Service (CTS) and PubChem Identifier Exchange Service (Barupal and Fiehn, 2017; Barupal et al., 2018). ClassyFire was used to automatically annotate metabolite classification with default parameters (Djoumbou Feunang et al., 2016).

### 2.4 Enrichment analysis and visualization

A heatmap of expression was drawn using R package pheatmap based on Z-scores-normalized values. Raw *p*-values were BH adjusted for multiple testing. The prcomp function of stats package in R performed PCA procedure to identify general trends in content changes. All identified and quantified protein annotations were considered as background datasets. Pathway enrichment analyses were performed in clusterProfiler package (version 4.4.4) (Wu et al., 2021). Visualizations of DEP and DAM intersecting sets were made through R package ggvenn and UpSetR (Conway et al., 2017). Overview bubble plots of GO (Gene Ontology) and KEGG (Kyoto Encyclopedia of Genes and Genomes) (Kanehisa and Goto, 2000; Kanehisa, 2019; Kanehisa et al., 2022) enriched terms were made by R package GOplot (Walter et al., 2015). Figures without special instructions were plotted by ggplot2 (Wickham, 2016). The results of enriched pathways, terms, and clusters with adjusted *p*-values ≤0.05 were considered significant.

### 2.5 Correlation analysis of proteins and metabolites

We examined correlations between protein data (six independent replicates) and metabolic profiles (ten independent replicates) to identify prospective proteins and metabolites that may modulate tail absorption. Because proteome and metabolome assays are destructive, direct comparison of the same samples was not possible. Therefore, datasets were randomly paired 1000 times, and the average spearman correlation between the values employed in two omics analyses was obtained. Only DEPs and DAMs from three groups were examined to limit the amount of calculations. Only the absolute value of correlations ≥0.8 were chosen in order to boost confidence levels. The correlation analysis scripts are accessible from Stephen A. Rolfe and Jurriaan Ton et al. (Cotton et al., 2019).

## 3 Results

**FIGURE 2:**
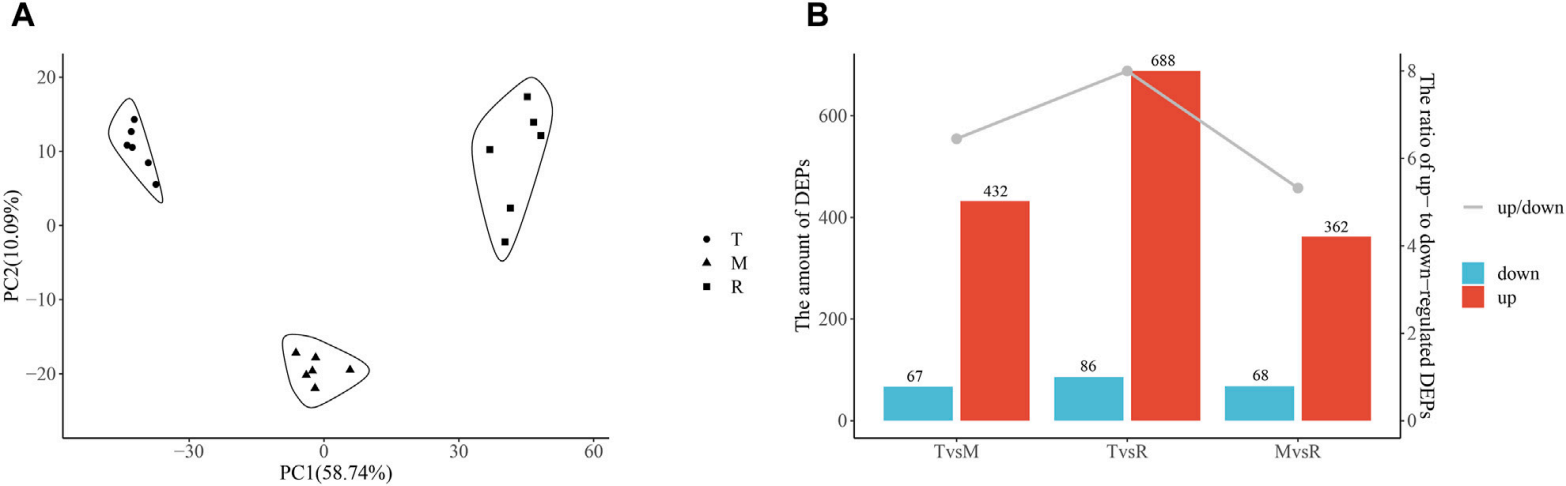
Principal component analysis (PCA) and differentially expressed proteins (DEPs). PCA of proteins **(A)**. Numbers of DEPs **(B)**. Column chart showing DEPs and the ratio of upregulated DEPs to downregulated DEPs (down/up). The ratio is shown on the second *Y*-axis, and the amount of DEP is shown on the first *Y*-axis. Tip (T), middle (M) and root (R).

**FIGURE 3:**
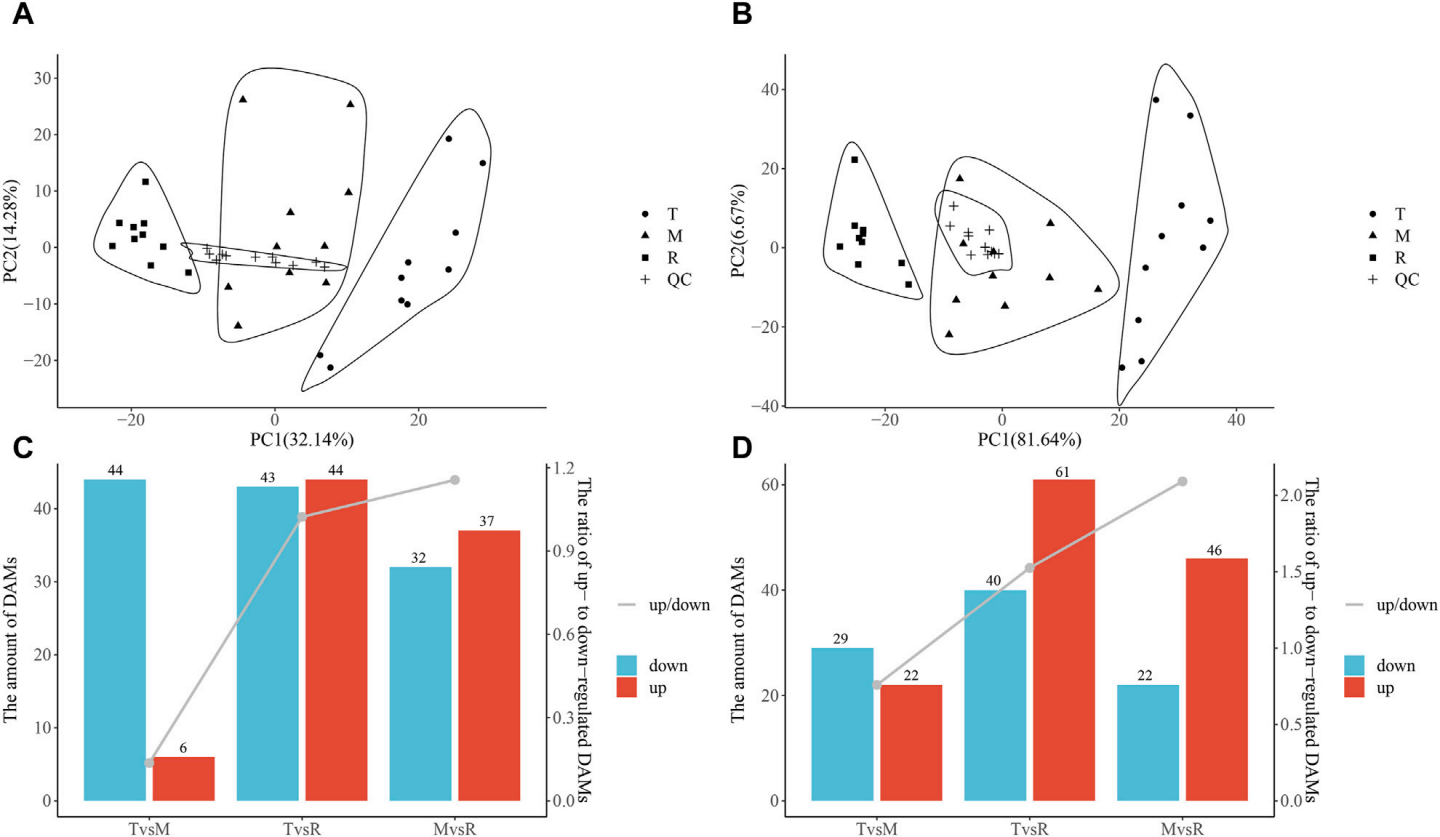
Principal component analysis (PCA) of metabolite and differentially accumulated metabolites (DAMs). PCA of metabolite negative ions **(A)** and metabolite positive ions **(B)**. Numbers of DAMs. Column chart showing DAMs and the ratio of upregulated DAMs to downregulated DAMs (down/up) in the negative ion mode **(C)** and in the positive ion mode **(D)**. The ratio is shown on the second *Y*-axis, and the amount of DAM is shown on the first *Y*-axis. Tip (T), middle (M), root (R) and quality control (QC) sample.

The results showed that proteins and metabolites had unique changes at each part and highly repeatability among duplicates (Figures 2A, 3A). From the tip to root of tail, PCA plot clearly demonstrated stepwise shifts show that three regions of tail underwent different metabolic changes.

### 3.1 The differentially expressed proteins

**FIGURE 4:**
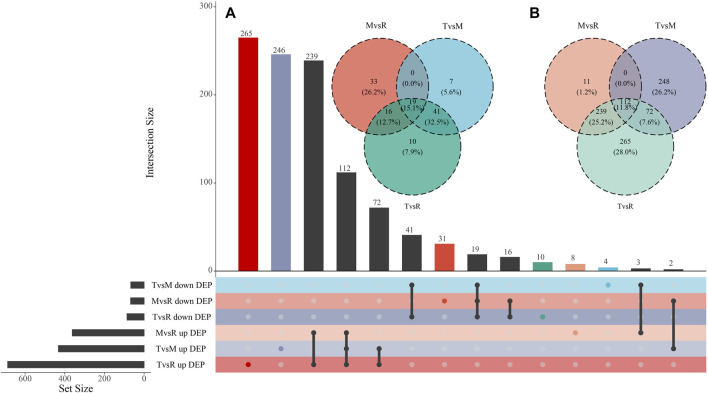
Visualizations of DEP (differentially expressed protein) intersecting sets through Venn and UpSet plot. **(A)** Venn plot of downregulated DEPs. **(B)** Venn plot of upregulated DEPs. The color of each group matches the one that is displayed in the lower left corner. tip (T), middle (M) and root (R). T vs. M, T vs. R and M vs. R.

After quantification and filter, 1857 unique proteins were detected in three groups (T vs. M, T vs. R, and M vs. R). 499 DEPs (432 upregulated, 67 downregulated), 774 DEPs (688 upregulated, 86 downregulated) and 430 DEPs (362 upregulated, 68 downregulated) were identified for T vs. M, T vs. R and M vs. R, respectively (Figure 2B; Supplementary Table S1). When a distal portion was compared with a proximal one, more upregulated DEPs were found. The comparison of T vs. R produced the largest significant variations among three comparisons. 1068 DEPs remained in union for DEPs of three comparison groups (Figure 4). 18 proteins showed continuously increase and, 102 proteins showed continuously decrease from distal to proximal portion, while two showed V-type and three showed inverted V-type curve variations (Supplementary Figures S1–S3).

### 3.2 Enrichment analysis

**FIGURE 5:**
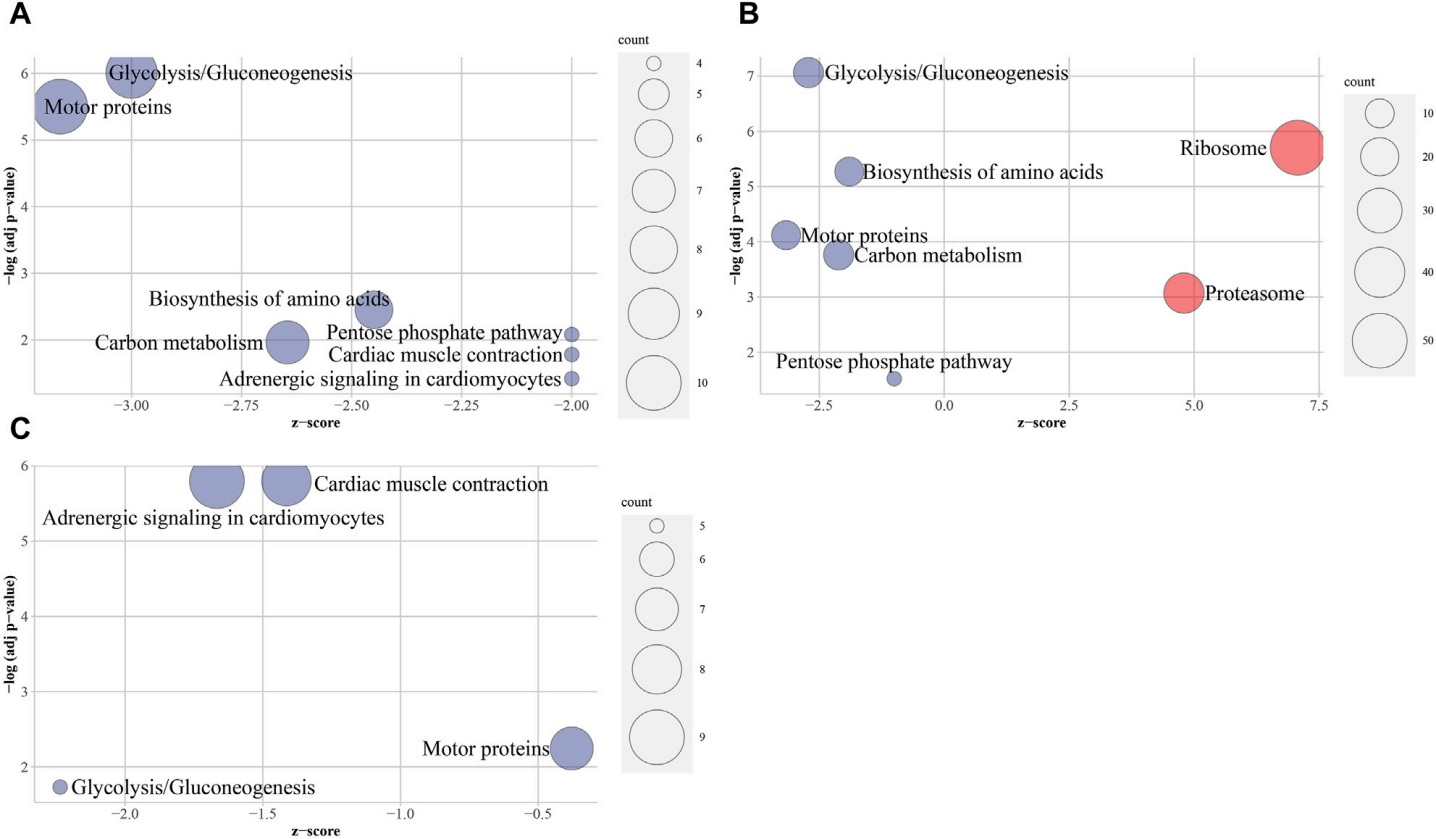
Overview bubble plots of KEGG (Kyoto Encyclopedia of Genes and Genomes) enriched terms. The z-score is assigned to the *x*-axis and the negative logarithm of the adjusted *p*-value to the *y*-axis. The area of the displayed circles is proportional to the number of genes assigned to the term and the color corresponds to the downregulated terms or upregulated terms. tip (T), middle (M) and root (R). **(A)** T vs. M, **(B)** T vs. R, **(C)** M vs. R.

Nine significantly enriched KEGG pathways were found in three groups (Figure 5; Supplementary Table S2). Downregulated DEPs were significantly enriched in two KEGG pathways “Glycolysis/Gluconeogenesis” and “Motor proteins”. Those from T vs. M and T vs. R were significantly enriched in three KEGG pathways “Biosynthesis of amino acids”, “Pentose phosphate pathway” and “Carbon metabolism”, while those from T vs. M and M vs. R were significantly enriched in two KEGG pathways of “Adrenergic signaling in cardiomyocytes” and “Cardiac muscle contraction”. On the other hand, upregulated DEPs from T vs. R were significantly enriched in two KEGG pathways of “Ribosome” and “Proteasome”. However, no significantly result remained in the upregulated DEPs from T vs. M and M vs. R. Interestingly, 23 DEPs (20S proteasome subunits and 26S proteasome regulatory subunits) (Supplementary Figure S4) and 50 DEPs (large subunit ribosomal proteins and small subunit ribosomal proteins) (Supplementary Figure S5) were involved in proteasome and ribosome pathways respectively.

### 3.3 GO enrichment analyses were conducted as complementary to KEGG

**FIGURE 6:**
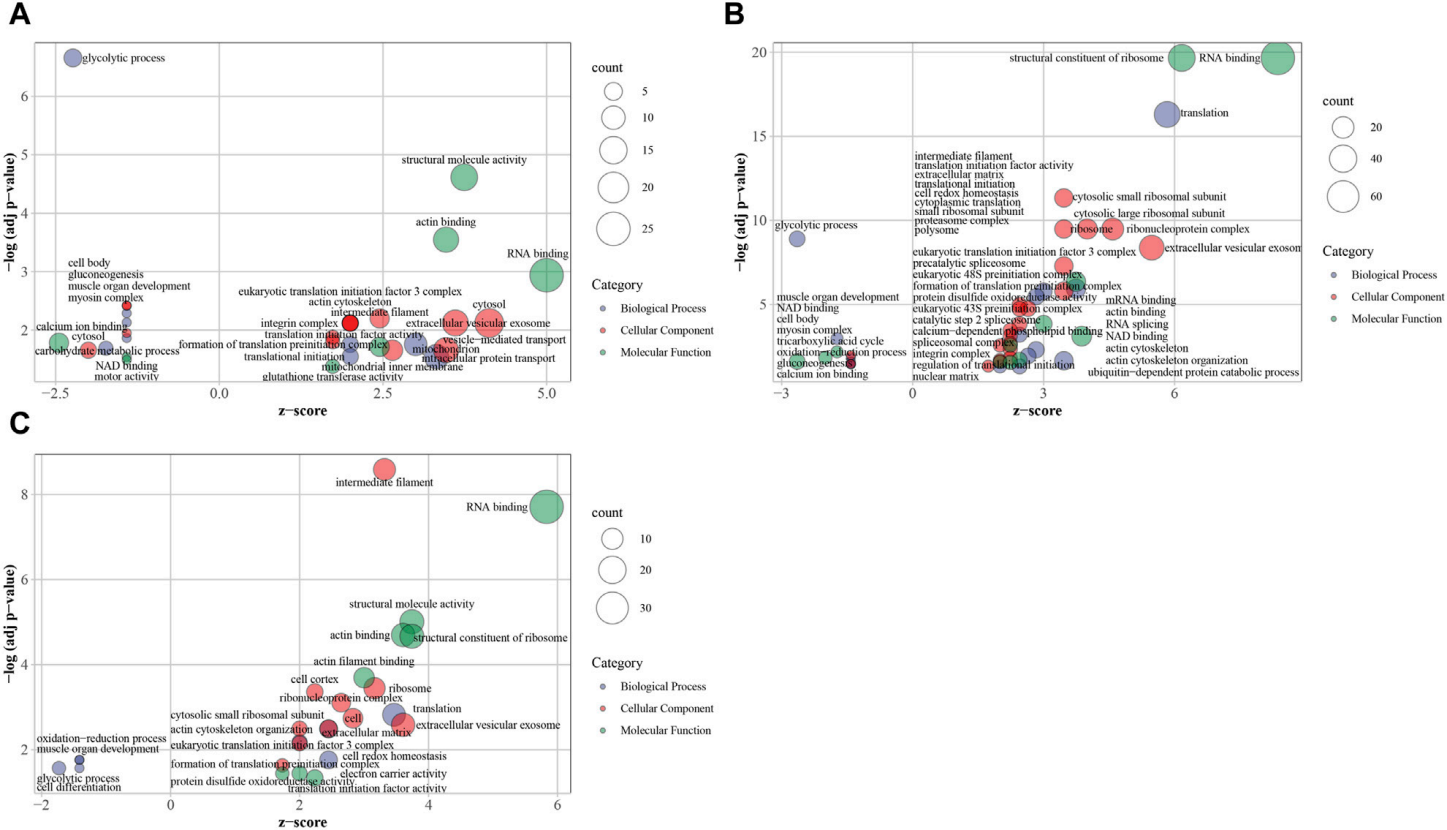
Overview bubble plots of GO (Gene Ontology) enriched terms. The z-score is assigned to the *x*-axis and the negative logarithm of the adjusted *p*-value to the *y*-axis. The area of the displayed circles is proportional to the number of genes assigned to the term and the color corresponds to the category (biological process, cellular component, molecular function). tip (T), middle (M) and root (R). **(A)** T vs. M, **(B)** T vs. R, **(C)** M vs. R.

74 significantly enriched GO terms were determined including 22 biological process terms, 33 cellular component terms and 19 molecular function terms in three groups (Figure 6; Supplementary Table S3). In downregulated DEPs of T vs. R and T vs. M groups, 11 shared terms included “GO:0006096 glycolytic process”, “GO:0007517 muscle organ development”, “GO:0006094 gluconeogenesis”, “GO:0010628 positive regulation of gene expression”, “GO:0044297 cell body”, “GO:0005829 cytosol”, “GO:0030027 lamellipodium”, “GO:0016459 myosin complex”, “GO:0051015 actin filament binding”, “GO:0051287 NAD binding”, “GO:0005509 calcium ion binding” which mostly related to energy and muscle. In downregulated DEPs of M vs. R group, four GO terms of “GO:0007517 muscle organ development”, “GO:0055114 oxidation-reduction process”, “GO:0006096 glycolytic process” and “GO:0030154 cell differentiation” were significantly enriched. In upregulated DEPs of T vs. R, T vs. M groups and M vs. R, 12 shared terms contain “GO:0001731 formation of translation preinitiation complex”, “GO:0034314 Arp2/3 complex-mediated actin nucleation”, “GO:0070062 extracellular vesicular exosome”, “GO:0005882 intermediate filament”, “GO:0005925 focal adhesion”, “GO:0005852 eukaryotic translation initiation factor 3 complex”, “GO:0003723 RNA binding”, “GO:0003743 translation initiation factor activity”, “GO:0051015 actin filament binding”, “GO:0005198 structural molecule activity”, “GO:0045296 cadherin binding” and “GO:0003779 actin binding”, which mostly related to translation.

### 3.4 The differentially accumulated metabolites

**FIGURE 7:**
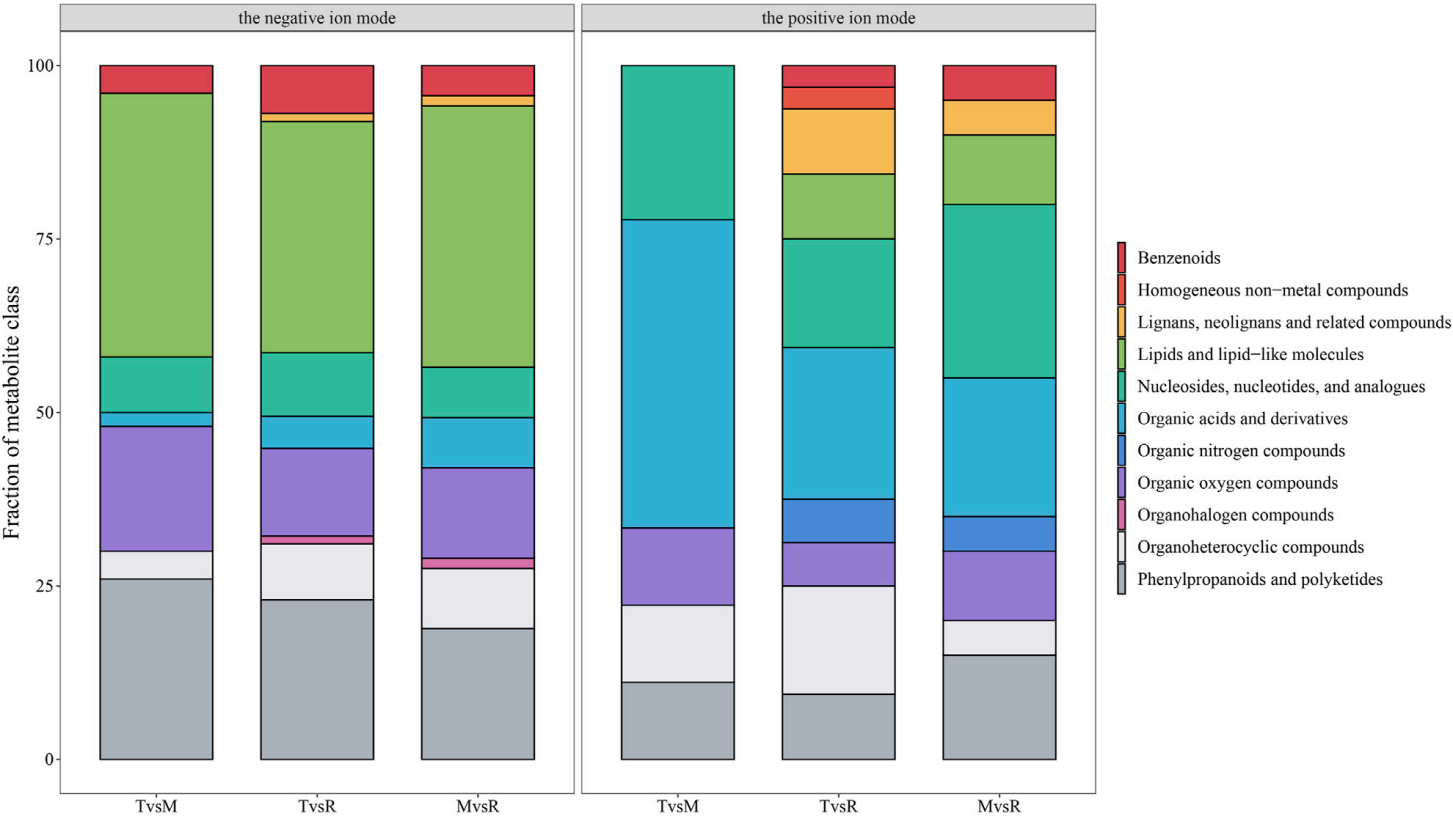
Fraction of the differentially accumulated metabolite (DAM) class in the three groups. The metabolite classification was automatically annotated by ClassyFire with default parameters. tip (T), middle (M) and root (R). T vs. M, T vs. R and M vs. R.

**FIGURE 8:**
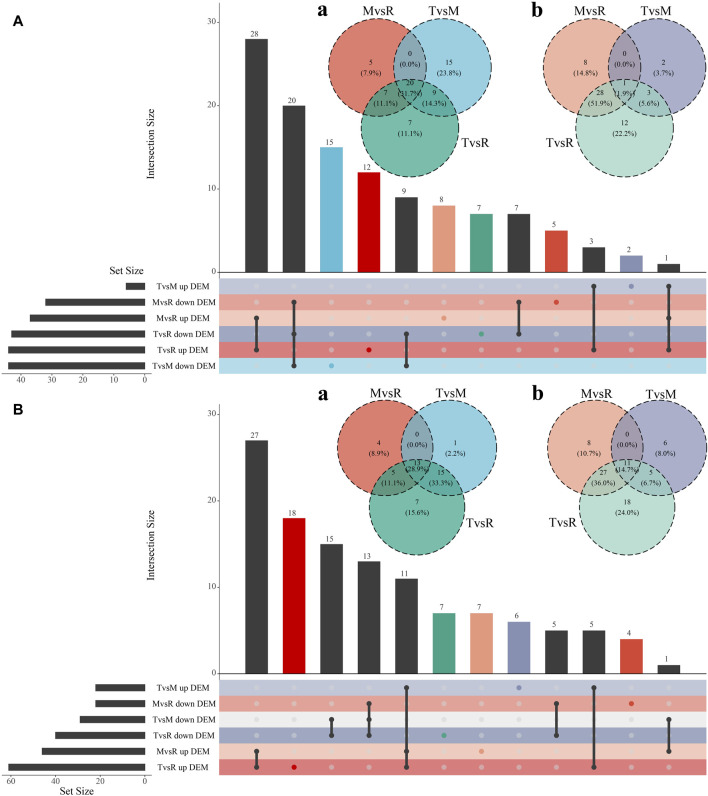
Visualizations of DAM (differential accumulated metabolite) intersecting sets through Venn and UpSet plot. Metabolites were acquired in the negative ion mode **(A)** and the positive ion mode **(B)**. (a) Venn plot of downregulated DAMs. (b) Venn plot of upregulated DAMs. The color of each group matches the one that is displayed in the lower left corner. tip (T), middle (M) and root (R). T vs. M, T vs. R and M vs. R.

The three tail parts were subjected to a nontargeted metabolomic analysis by high-resolution LC-MS in order to characterize the chemicals objectively on a broad scale. QC samples were closely clustered in the center of PCA plot, demonstrating robust repeatability of metabolite extraction and metabolomic analysis as well as the stability of instrument (Figures 3A, B). Using MS-DIAL software, 647 and 2226 metabolites with annotation were identified in negative and positive ion mode respectively. In the negative ion mode, 50 DAMs (6 upregulated, 44 downregulated), 87 DAMs (44 upregulated, 43 downregulated), and 69 DAMs (37 upregulated, 32 downregulated) (Figure 3C) were confirmed for T vs. M, T vs. R and M vs. R, respectively. 51 DAMs (22 upregulated, 29 downregulated), 101 DAMs (61 upregulated, 40 downregulated), and 68 DAMs (46 upregulated, 22 downregulated) were identified for T vs. M, T vs. R and M vs. R in the positive ion mode, respectively (Figure 3D). 230 metabolites were kept in union of DAMs in three comparison groups (Supplementary Table S4). Most of DAMs were classed as purine ribonucleoside monophosphates, 2-acyl-sn-glycero-3-phosphocholines, flavonoid glycosides, hybrid peptides, pyranones and derivatives, aminoalcohols, histidine and derivatives, iridoid O-glycosides. These DAMs span over eleven categories (benzenoids, homogeneous non-metal compounds, lignans, neolignans and related compounds, lipids and lipid-like molecules, nucleosides, nucleotides, and analogues, organic acids and derivatives, organic nitrogen compounds, organic oxygen compounds, organohalogen compounds, organoheterocyclic compounds, phenylpropanoids and polyketides) (Figure 7). 45 DAMs were shared in three comparisons (Figure 8), and mainly comprising purine ribonucleoside monophosphates, flavonoid glycosides, hexose phosphates, histidine and derivatives, hybrid peptides, iridoid O-glycosides, P-benzoquinones class. One metabolite displayed inverted V-type curve changes, while 13 metabolites constantly increased and 12 metabolites constantly decreased from the distal to proximal section. (Supplementary Figures S6–S8).

### 3.5 ChemRICH analysis

**FIGURE 9:**
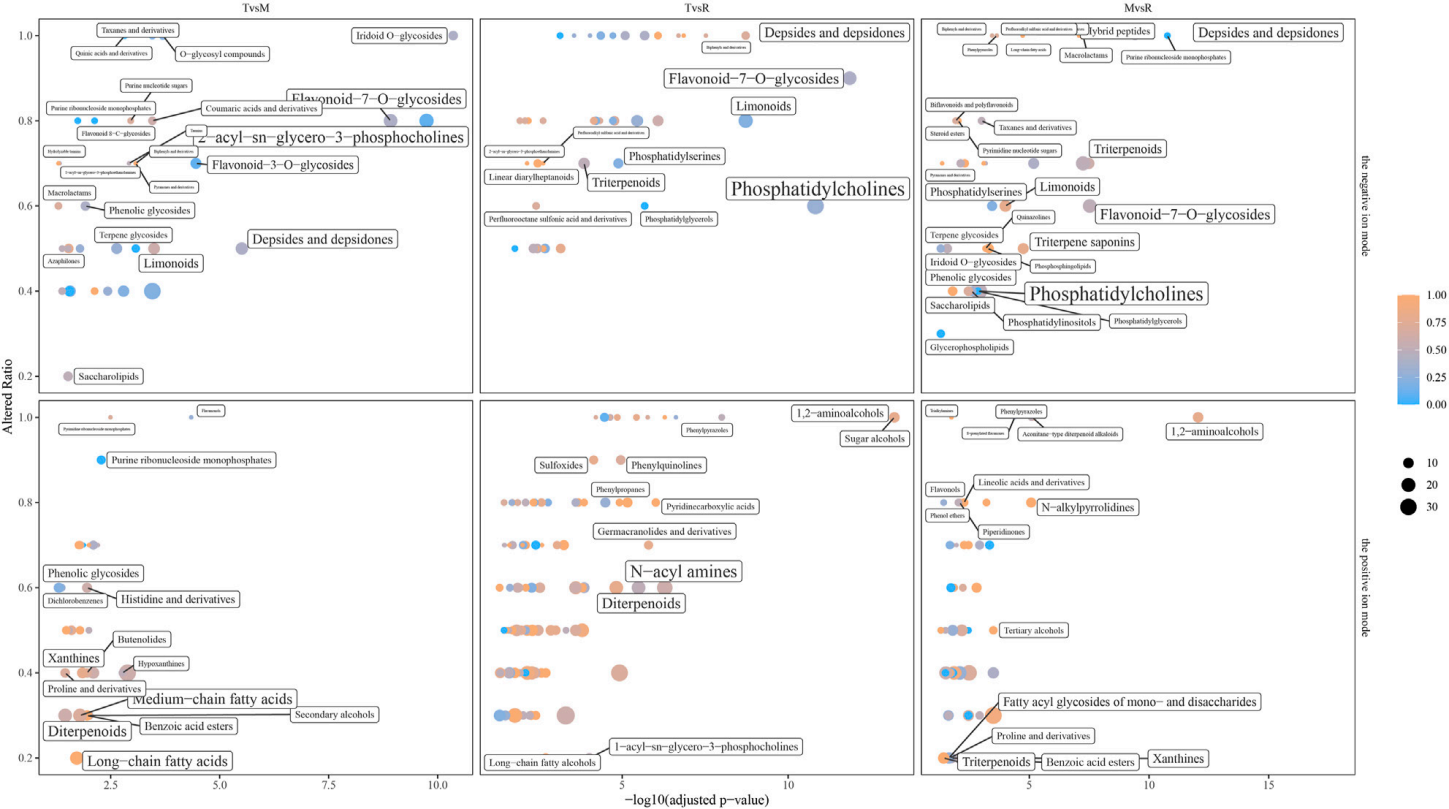
Chemical similarity enrichment analysis (ChemRICH) set enrichment statistics plot. Point size corresponds to the count of metabolites in each cluster set. Point color shows that proportion of the increased metabolites where red means high and blue means low number of upregulated compounds. tip (T), middle (M) and root (R).

A total of 185 significantly enriched metabolite sets (T vs. M: 65, T vs. R: 171 and M vs. R: 95) were found by ChemRICH analysis, mostly contain purine ribonucleoside monophosphates, depsides and depsidones, flavonoid-7-O-glycosides, hybrid peptides, O-glycosyl compounds, phenolic glycosides, pyranones and derivatives, fatty acyl glycosides of mono- and disaccharides, saccharolipids, triterpenoids (Figure 9; Supplementary Table S5).

### 3.6 Integrative analysis

**FIGURE 10:**
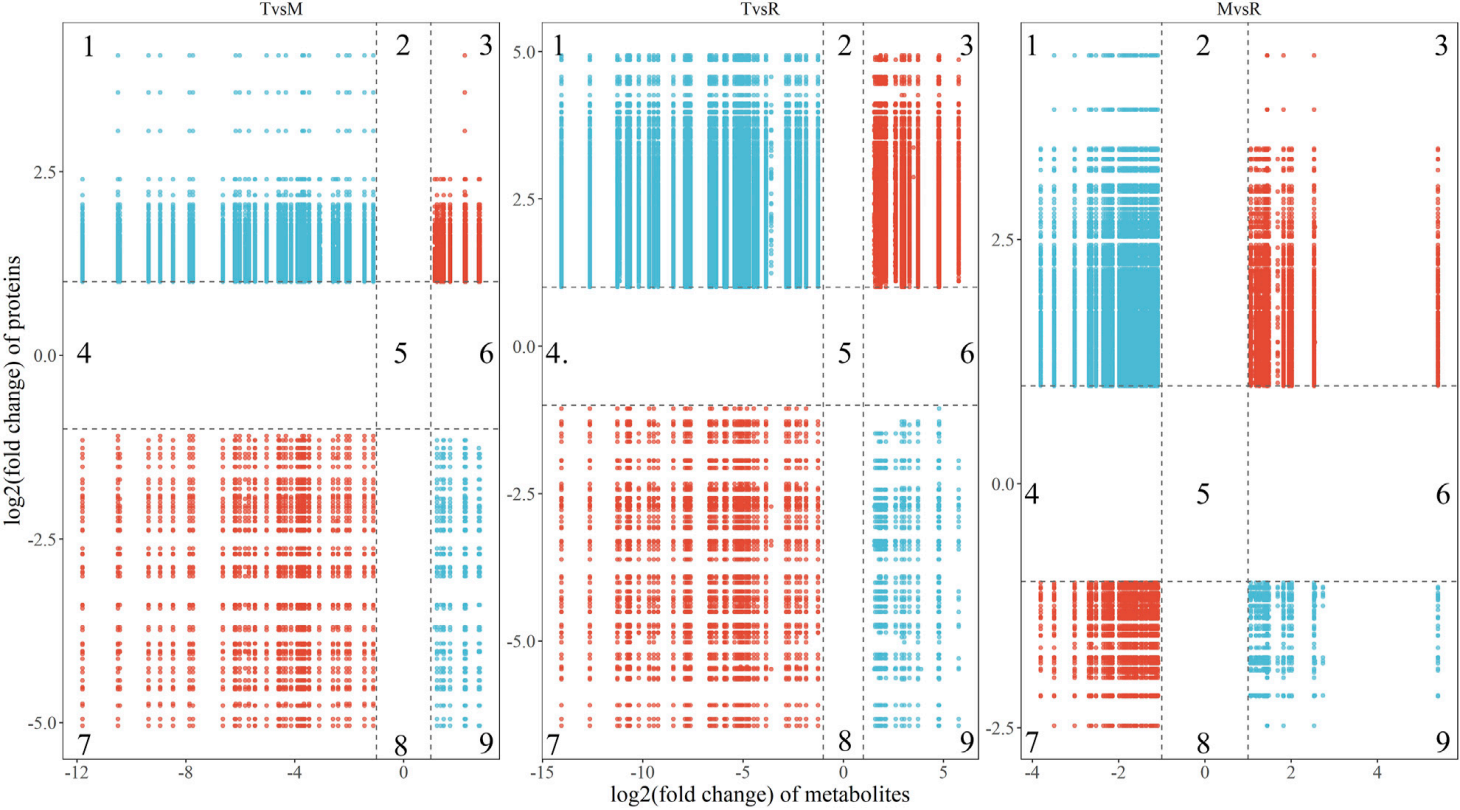
Nine-quadrant diagrams show the correlation (the absolute value of correlation coefficient >0.8) of differentially accumulated metabolite (DAM) and differentially expressed protein (DEP) in three groups (T vs. M, T vs. R and M vs. R). The *Y*-axis indicates the log_2_ (fold change) of proteins, and the *X*-axis indicates the log_2_(fold change) of metabolites. The subgraph was divided into nine quadrants by black dashed lines. Quadrant three and seven represented DEP and DAM with positive correlation; quadrant one and nine represent DEP and DAM with negative correlation; quadrant 2, 4, 5, six and eight represent not DEP and DAM and no correlations between genes and metabolites. tip (T), middle (M) and root (R). T vs. M, T vs. R and M vs. R.

**FIGURE 11:**
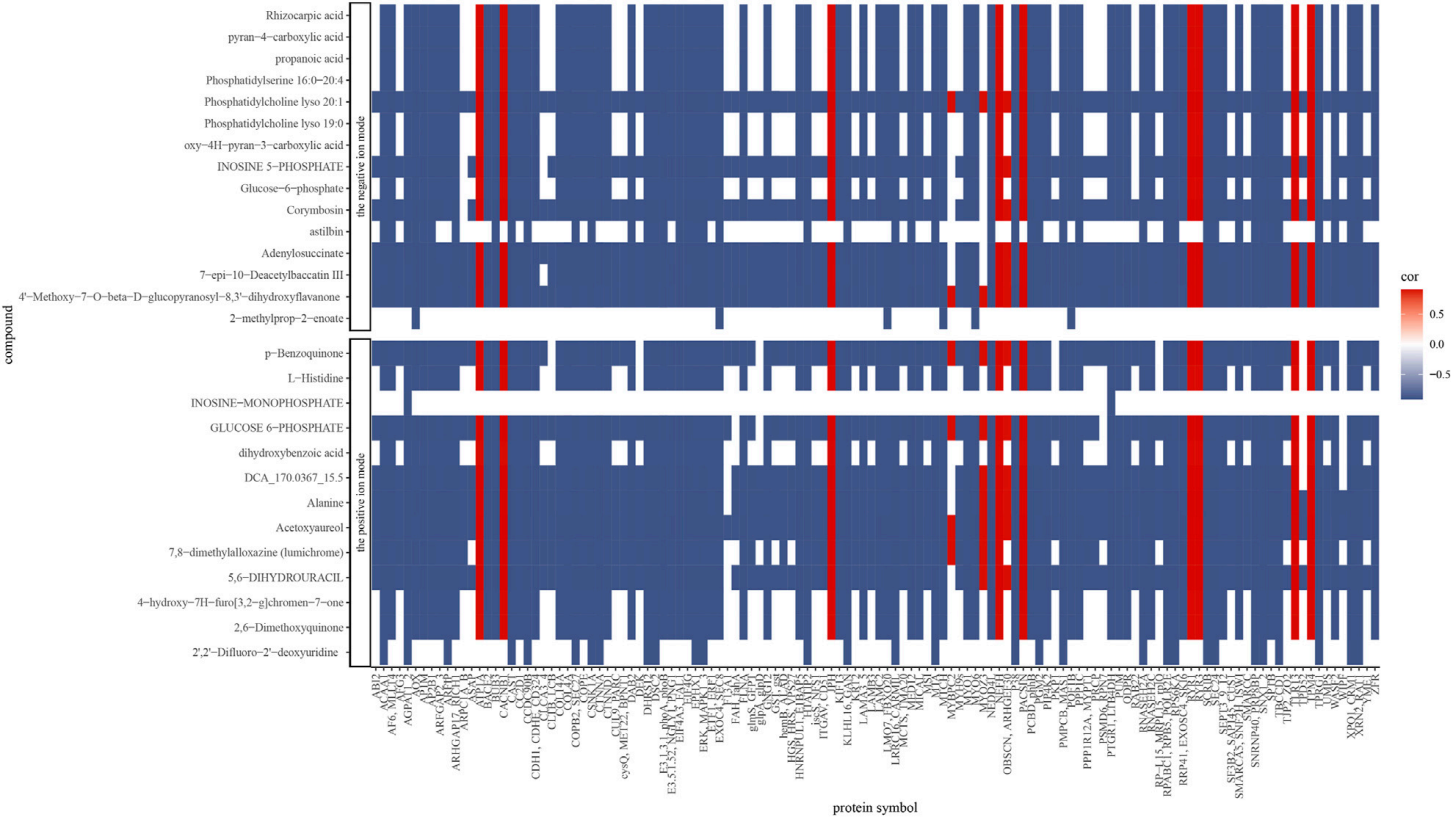
Spearman correlations between proteins and metabolites abundance. Only correlations ≥0.9 are presented (positive correlations: red; negative correlations: blue).

The correlations of all DEPs and DAMs in each comparison were showed in the nine-quadrant diagrams (Figure 10). These correlations involve 865 proteins and 98 metabolites (Supplementary Table S6). While DAMs and DEPs in quadrants one and nine are negatively correlated, those in quadrants three and seven are positively correlated. The absolute values of correlation coefficient of 134 proteins and 28 metabolites were above 0.9 (Figure 11; Supplementary Table S7). The 28 metabolites mainly related to purine ribonucleoside monophosphates, 2-acyl-sn-glycero-3-phosphocholines and hexose phosphates. The nine enriched GO terms (*p*-value <0.05) in the 134 proteins were intracellular protein transport (GO:0006886), MAP kinase activity (GO:0004707), actin filament binding (GO:0051015), cadherin binding (GO:0045296), NAD binding (GO:0051287), RNA binding (GO:0003723), actin binding (GO:0003779), protein serine/threonine kinase activity (GO:0004674) and structural molecule activity (GO:0005198). The eight enriched KEGG terms (*p*-value <0.05) in the 134 proteins were endocytosis (ko04144), adherens junction (ko04520), adrenergic signaling in cardiomyocytes (ko04261), apelin signaling pathway (ko04371), regulation of actin cytoskeleton (ko04810), MAPK signaling pathway (ko04010), vascular smooth muscle contraction (ko04270) and focal adhesion (ko04510).

## 4 Discussion

The proteomic and metabolomic data can provide the biochemical landscape for further exploration of molecular mechanism of tail resorption in *N*. *pleskei*. Higher metabolism was indicated by more upregulated proteins in the distal portions of tail.

### 4.1 Response to thyroid hormone

The main metabolic pathways are all impacted by thyroid hormones (THs). Their most evident and well-known effect is raising basal energy expenditure through effects on lipid, protein, and carbohydrate metabolism (Pucci et al., 2000). The levels of plasma TH in *Xenopus liaevis* tadpole were elevated during metamorphosis climax (Shi et al., 1996). T3 primarily affects biological metabolism via TH receptors (TR) in all vertebrates (Cheng et al., 2010; Krieger et al., 2019; Shi, 2021). TR double knockout (TRDKO) in *Xenopus tropicalis* tadpoles with or without T3 treatment revealed that TR is extremely important to coordinate the effects of T3 on metamorphosis (Wang et al., 2023).

THs can operate in two ways (Moeller and Broecker-Preuss, 2011). First, classically through the nuclear TR α and TRβ residing on thyroid hormone response elements (TREs) in the promoter of TH target genes, they can result in gene induction as described above. Second, non-classically through two pathways: via site one of integrin αVβ3 they can induce PI3K (phosphatidylinositol-4,5-bisphosphate 3-kinase) in the cytoplasm and nucleus; via site two of αVβ3 they can activate the MAPK pathway.

The common integrin ligands are proteins, however THs, a small molecule, can also bind to an integrin (Bergh et al., 2005). Integrin alpha V (ITGAV, CD51, K06487) and integrin beta 3 (ITGB3, CD61, K06493) are present in genome of *X. tropicalis*, whereas integrin alpha 5 (ITGA5, CD49e, K06484) is absent. In our investigation, ITGB3, ITGA5 and ITGAV were identified as DEPs (Supplementary Figure S9). ITGAV and ITGA5 are members of two clusters of differentiation (CD) and have distinct functions. ITGAV and ITGB3 forms heterodimers αVβ3 (Bristow et al., 2020). We recognized a fact that TRα and TRβ were not detected in regressing tail. One of the reasons may be that the protein file of *N*. *parkeri* genome we used does not include TRα and TRβ, because we did not find TRα and TRβ in the functional annotation file of *N. parkeri* genome. In addition, the protein files of *X. tropicalis* and *X. laevis* genomes were used to search in DIA-NN software, the result of identified proteins does not include TRα and TRβ either. TRα and TRβ of *X. tropicalis* and *X. laevis* were searched against to protein of *N. parkeri* genome by *blastp* software and no match was turned out. *N. pleskei* may not contain TRα and TRβ. More researches are needed to answer this question. Actually, the mitogen-activated protein kinase 1/3 (K04371, ERK, MAPK1/3) in the MAPK pathway were upregulated in tip tail of *N. pleskei*. These results suggest that non-classical action of TH works in *N. pleskei*. Consequently, the higher expression of integrin αVβ3 in the tail tip means tip portion has elevated sensibility to TH during the metamorphosis in *N. pleskei*.

98 DAMs (37 positive and 61 negative) strongly related to integrin alpha V, integrin beta 3, MAPK (absolute value of correlations ≥0.8) were grouped into 56 categories. (Supplementary Figures S10, S11). The 37 positively and 61 negatively correlated compounds were assigned into eight common categories, such as “benzenoids”, “lignans, neolignans and related compounds”, “lipids and lipid-like molecules”, “nucleosides, nucleotides, and analogues”, “organic acids and derivatives”, “organic oxygen compounds”, “organoheterocyclic compounds”, and “phenylpropanoids and polyketides”. Among these, L-histidine and glucose-6-phosphate stand out. It is worth noting that nucleosides and nucleotides may be dramatically degraded in the distal portion, which is trigged by THs, and transport to the other parts of body for reuse.

### 4.2 Space pattern of muscle regression

Thyroid hormone-induced tail regression is a multi-step process that involves the death of muscle cells and many other types of cells, the migration of macrophages, and the absorption of cell debris (Nakajima et al., 2005). One of the organ remodeling processes during metamorphosis is tail absorption (Brown and Cai, 2007). The tadpole absorbs tail that is as twice long as its trunk within a matter of days, and all the time its locomotive function is kept under constant control as its driving force shifts from its tail to hindlimbs (Yaoita, 2019).

The expression of two proteins lysosomal Pro-X carboxypeptidase (PRCP, K01285) and cathepsin A (carboxypeptidase C, CTSA, CPYK, 13289) which related to lysosome were significantly high in the tip of tail (Supplementary Figure S12). Ten proteins in motor protein pathway (ko04814) were significantly less expressed in the distal portion compared to proximal portion. Meanwhile, two GO terms of “muscle organ development (GO:0007517)” and “glycolytic process (GO:0006096)” were also significantly downregulated in the distal portions. One major metabolic process that produces ATP in cells is glycolysis (Chandel, 2021). The rate of glycolysis was directly correlated with muscle contraction and relaxation (Helmreich and Cori, 1965). In the tail of *R. omeimontis* tadpoles at metamorphic climax, the metabolic flux throughout glycolysis, Ca^2+^-ATPases, and muscle creatine kinases which are the primary ATP consumers were downregulated (Zhu et al., 2020). These results indicate that the ability of energy production in muscle was reduced, and that the growth of muscles was dismissed in the distal portion of tail. In addition, the augmented function of lysosome can aid the degradation of muscle and other tissues. Nine GO terms were found to be significantly downregulated in both T vs. R and T vs. M groups. Four of them “GO:0007517 muscle organ development”, “GO:0016459 myosin complex”, “GO:0051015 actin filament binding”, and “GO:0005509 calcium ion binding” are involved in functions of muscle. This further implies that the loss of aforementioned functions begins at the tip of tail. This was also consistent with our observation that tadpoles prefer to remain motionless during metamorphosis climax. Similarly, in *X*. *laevis* tadpoles, more than half of the genes for the tricarboxylic acid pathway and the cytoplasmic glycolytic enzymes were downregulated in tail muscle during climax of metamorphosis (Das et al., 2006).

The structure and physiological processes of cardiac and skeletal muscle are comparable (Du et al., 2014; Weizhao et al., 2021). The five proteins, P-type Ca^2+^ transporter type 2A (ATP2A, K05853), myosin light chain 4 (MYL4, K12750), tropomyosin 1 (TPM1, K10373), fast skeletal myosin light chain 2 (MYLPF, K12758) and actin (ACTC1, K12314) related to muscle contraction, adrenergic signaling in cardiomyocytes (ko04261) and motor proteins (ko04814) were constantly increased from distal to proximal portion of tail. As predicted, the function of muscular contraction may vanish gradually from the tip of tadpole tail. Additionally, in the DEP set which constantly decreased from proximal to distal portion, three crucial enzymes, triosephosphate isomerase (K01803 TPI, tpiA), L-lactate dehydrogenase (LDH, K00016) and pyruvate kinase (PK, K00873) in the glycolysis/gluconeogenesis (ko00010) pathway may also contribute the shortage of energy supply in the tail tip. In the DEP set which constantly increased from proximal to distal portion, ten proteins, actin related protein 2/3 complex subunit 2 (ARPC2, K05758), actin related protein 2/3 complex subunit 4 (ARPC4, K05755), charged multivesicular body protein 4A/B (CHMP4A_B, SNF7, VPS32A_B, K12194), clathrin heavy chain (CLTC, K04646), EH domain-containing protein 4 (EHD4, K12477), endophilin-B1 (SH3GLB1, K11248), epsin (EPN, K12471), Ras-related protein Rab-11A (RAB11A, K07904), Ras-related protein Rab-22 (RAB22, K07891) and WAS/WASL-interacting protein (WIPF, K19475) in the endocytosis (ko04144) pathway indicate that the tail tip experienced a more active cell breakdown. Five proteins, ARPC2, ARPC4, actinin alpha 1/4 (ACTN1_4, K05699), cytoplasmic FMR1 interacting protein (CYFIP, K05749), integrin alpha V (ITGAV, CD51, K06487), integrin beta 4 (ITGB4, CD104, K06525) and villin two (ezrin, VIL2, K08007) in the regulation of actin cytoskeleton (ko04810) pathway suggest that cytoskeletal remodeling occurred at the tail tip.

### 4.3 The murder and suicide models

Muscle cells in the tail perform both the suicide and murder procedures throughout the entire process of tail shortening in *X. laevis* tadpole (Nakajima et al., 2005). In present study, matrix metalloproteinase-3 (MMP3, stromelysin-1, progelatinase, K01394) exhibited higher expression level in the tail tip (Supplementary Figure S12). It makes up the main proteolytic enzyme group that breaks down the extracellular matrix constituents (Saus et al., 1988). Stromelysin-3 (MMP 11) from matrix metalloproteinase (MMP) family was proved to play an important role in *X*. *laevis* tadpole tail murder model (Patterton et al., 1995; Damjanovski et al., 1999; Ishizuya-Oka et al., 2000; Mathew et al., 2009; Mathew et al., 2010). Both MMP11 and MMP3 are members of MMP family, which function as extracellular endopeptidases in vertebrate tissues and are similar to interstitial collagenase. They can degrade extracellular macromolecules (Jackson et al., 2010).And MMP3 was involved in *N. pleskei*. This may be due to species heterogeneity and plateau specialization.

On the other hand, two GO terms “GO:0043161 proteasome-mediated ubiquitin-dependent protein catabolic process” and “GO:0006511 ubiquitin-dependent protein catabolic process” were significantly enriched in upregulated DEPs in the T vs. R. Meanwhile, programmed cell death six-interacting protein (PDCD6IP, ALIX, RIM20, K12200) was significantly upregulated in the tail tip. It is proved that the PDCD6IP is involved in the processes of apoptosis (Blum et al., 2004; Hemming et al., 2004). The overexpression of PDCD6IP can induce apoptosis (Trioulier et al., 2004; Anne-Laure et al., 2006). This imply that the tail resorption of *N. pleskei* tadpole exhibits characteristics of both suicide and murder patterns.

Metabolites from breakdown of proteins and other macromolecules were relatively abundant at the tail tip. For example, elevated levels of long-chain fatty acids (tetradec-5-ynoic acid), medium-chain fatty acids (heptanoic acid), L-alpha-amino acids (L-2,3-diaminopropionic acid), histidine and derivatives (ergothioneine) and purines (adenine and guanine) were observed in the tail tip.

### 4.4 Ribosome and proteasome KEGG pathways

Ribosomes are responsible for the synthesis of proteins, including proteases, while the proteasomes are involved in the degradation of proteins. Our results indicated that both ribosome and proteasome KEGG pathways were identified in upregulated DEPs of T vs. R. The substantially upregulated enrichment GO in T vs. R also showed terms linked to translation and protein catabolic process (Supplementary Figure S13). In *Rana omeimontis*, downregulated transcription of aminoacyl-tRNA synthetase, ribosomal components, and aminotransferases suggests that protein synthesis during the metamorphic climax (G43) was suppressed in the whole tail of tadpoles (Zhu et al., 2020). This result was obtained from the comparison between different developmental stages while our result came from the comparison between different space of the tail. In the tail tip of *X*. *tropicalis* tadpoles, corticosterone (CORT) increased the expression of seven genes in the ribosome GO term, and CORT-TH synergy enhanced the expression of two genes in the proteasome complex (Kulkarni and Buchholz, 2012). Fourteen genes, including proteolytic enzymes in proteasome, were upregulated in dying muscle fibers in the tail of *X*. *laevis* tadpoles (Das et al., 2006).

### 4.5 Chemical similarity enrichment analysis (ChemRICH)

How metabolism reacts to genetic, phenotypic, and environmental factors is a central question in biology that could be answered by metabolomics. However, due to low abundance or limitations imposed by chemistry and biology, many intermediates in different pathways are missed by metabolomics experiments. This issue also exists in our experiment. Additionally, many identified metabolites were not annotated to the known metabolic pathways, especially for complex metabolites. ChemRICH can compensates for this deficiency in certain extent. Chemical classification of all identified compounds in a metabolome research can give significantly relevant biological importance. In present study, relatively fewer metabolites (5.0%) were discovered to have significant alteration than proteins (57.5%). In the data analysis, we used all of the substances that MS-DIAL software had successfully identified. With strict screening standard, only a small number of metabolites remained as significant variated DAMs.

A number of classes of DAMs was noted, including elevations in “purine ribonucleoside monophosphates” and “flavonoid O-glycosides”, reductions in “pyrimidine”, “amino acids and derivatives”, “pyrimidine and derivatives”, and major fatty acids, most notably medium-chain and long-chain fatty acids. Opposite variation was observed in purine and pyrimidine derivatives.

Regeneration which include reprogramming is the process of replacing harmed, ill or aged tissues (Jopling et al., 2011). Uridine is identified as one of regeneration-related metabolite across ages, tissues and species (axolotls *Ambystoma mexicanum*, deer antler, cynomolgus monkeys, C57BL/6 J mice and human). It was proved that higher regenerative capability was connected with active pyrimidine metabolism, amino acid metabolism and fatty acid metabolism (Liu et al., 2022). The resorption of the tail of *N. pleskei* may closely associated with regeneration. In present study, nucleotide, amino acid and fatty acid metabolism appeared more prominent in the tip of tail during resorption. The root tail of *N. pleskei* was found to contain more uridine, but thymine was not a DAM across three tail sections. The expression levels of three pyrimidine nucleotide sugars, uridine diphosphoglucuronic acid, uridine diphosphoglucose and uridine 5'−diphospho−N−acetylgalactosamine were higher in the tail tip than that in the middle and root. This indicates the tail tip was continually healing as it was shortening and the tissues was reabsorbed. The structural components of DNA and RNA, pyrimidine and purine, offer essential substrates for vital biological processes during metamorphic climax.

Meanwhile, amino acid maybe catabolized for energy in the tail. The tadpoles at metamorphic climax will experience limitation of energy. Amino acid contributed more to energy supply in liver of *Rana omeimontis* tadpoles during the metamorphic climax (Zhu et al., 2020). Fatty acid elongation and desaturation, as well as the production of bioactive lipid, were promoted in *R. omeimontis* apoptotic tail during metamorphic climax (Zhu et al., 2020). In present work, our result indicted the concentration of some amino acids and their derivatives was higher in the tip than middle and root tail. These amino acids may come from the proteolysis and be transported to other part of the body for reuse.

### 4.6 Protein and metabolite linkage

Integrative analysis was performed by spearman correlation. We identified 28 metabolites which displayed a root-to-tip decreasing trend with 121 negatively correlated proteins and 12 positively correlated proteins (Supplementary Figures S14–S16). Within the positively correlated set, five proteins, ryanodine receptor 1 (RYR1, K04961) and ryanodine receptor 3 (RYR3, K04963) in calcium signaling pathway (ko04020), and voltage-dependent calcium channel beta-1 (CACNB1, K04862), tropomyosin 4 (TPM4, K10375) and sodium/potassium-transporting ATPase subunit alpha (ATP1A, K01539) in cardiac muscle contraction (ko04260) imply gradual weakening of muscle function. In the negatively correlated set, a total of twenty proteins in endocytosis (ko04144) and regulation of actin cytoskeleton (ko04810) pathways suggest that more severe cell degradation occurred in the tail tip, which is consistent with the result mentioned above (Supplementary Figure S17). Among the 28 identified metabolites, glucose 6-phosphate (C00092), inosine 5-phosphate (C00130), adenylosuccinate (C03794) and alanine (C00041) are energy-related, supporting the muscle cells with energy and facilitating the action of tail root muscles.

## 5 Conclusion

By combining proteomic and metabolomic approaches, we provide fresh insight into the mechanisms underlying ordered resorption from the tip to root of tail in *N. pleskei* tadpole metamorphism. High-expressed αVβ3 in the tip can enhance the response sensibility to thyroid hormone. Both the suicide model and murder model can be applied to the tail resorption in this species. Muscle regression showed a space pattern with stronger regression in the distal and weaker one in the proximal portion. Both protein synthesis and proteolysis were active in the tip tail. The products from the breakdown of tissues can be transported to other parts of the body and used as building blocks or energy sources. This study offers a solid foundation for subsequent researches aiming to explore the portion specific resorption of the tadpole tail and related regulation of proteins and metabolites in greater depth.

## Data Availability

The datasets presented in this study can be found in online repositories. The names of the repository/repositories and accession number(s) can be found below: https://ngdc.cncb.ac.cn/omix, OMIX005159; https://ngdc.cncb.ac.cn/omix, OMIX005163; https://ngdc.cncb.ac.cn/omix, OMIX005164.
