# Supplementary material for: Integrative proteome and metabolome analyses reveal molecular basis of the tail resorption during the metamorphic climax of Nanorana pleskei
Source: Front Cell Dev Biol. 2024 Aug 19;12:1431173. doi: 10.3389/fcell.2024.1431173 (PMC11366584; doi:10.3389/fcell.2024.1431173)
Supplement: Supplementary file 1 [file DataSheet2.PDF]

**Supplementary Text S1 MS-DIAL parameter settings**

**Ionization type parameters**

Soft Ionization

**Separation type parameters**

Chromatography

**MS method type**

Conventional LC/MS or data dependent MS/MS

**Data type (MS1)**

Profile data

**Data type (MS2)**

Centroid data

**Data collection parameters**

Retention time begin 0 min; Retention time end 100 min; Mass range begin 70 Da; Mass range end 1050 Da; MS/MS mass range begin 0 Da; MS/MS mass range end 2000 Da.

**Peak detection parameters**

Minimum peak height 10000 amplitude; Mass slice width 0.1 Da; Smoothing method Linear weighted moving average; Smoothing level 2 scan; Minimum peak width 5 scan.

**MS2Dec parameters**

Sigma window value 0.5; MS/MS abundance cut off 10 amplitude; Exclude after precursor ion True

**Identification parameters**

MSP file: MSMS-Public-Neg-VS17.msp for negative ion mode, MSMS-Public-Pos-VS17.msp for positive ion mode; Retention time tolerance 100 min; Accurate mass tolerance (MS1) 0.01 Da; Accurate mass tolerance (MS2) 0.05 Da; Identification score cut off 60%; Only report the top hit.

**No Text file and post identification (retention time and accurate mass based) setting**

**Adduct**

$[M-H]^-$ and $[M+HCOO]^-$ for negative ion mode,

$[M+H]^+$, $[M+CH_3OH+H]^+$, $[M+ACN+H]^+$, $[M+H-H_2O]^+$ and $[M+H-2H_2O]^+$ for positive ion mode

**Alignment parameters**

Reference file: QC; Retention time tolerance: 0.05 min; MS1 tolerance: 0.025 Da; Retention time factor: 0.5; MS1 factor: 0.5; Peak count filter: 25 %; N% detected in at least one group: 16.7 %
