# Supplementary material for: Integrative proteome and metabolome analyses reveal molecular basis of the tail resorption during the metamorphic climax of Nanorana pleskei
Source: Front Cell Dev Biol. 2024 Aug 19;12:1431173. doi: 10.3389/fcell.2024.1431173 (PMC11366584; doi:10.3389/fcell.2024.1431173)
Supplement: Supplementary file 5 [file DataSheet1.PDF]

Supplementary Figure S1. Expression boxplot of 18 uptrendly expressed proteins in the three portions of *Nanorana pleskei*. The protein symbol and the KEGG orthology (ko) ID are listed outside and inside of bracket on the top of the subgraph. tip (T), middle (M) and root (R).

Supplementary Figure S2. Expression boxplot of 102 downtrendly expressed proteins in the three portions of *Nanorana pleskei*. The protein symbol and the KEGG orthology (ko) ID are listed outside and inside of bracket on the top of the subgraph. tip (T), middle (M) and root (R).

Supplementary Figure S3. Expression boxplot of 3 inverted V type and 2 positive V type expressed proteins in the three portions of *Nanorana pleskei*. The protein symbol and the KEGG orthology (ko) ID are listed outside and inside of bracket on the top of the subgraph. tip (T), middle (M) and root (R).

Supplementary Figure S4. Boxplots of the proteins related to proteasome (ko03050).

Supplementary Figure S5. Boxplots of the proteins related to Ribosome (ko03010).

Supplementary Figure S6. Expression boxplot of 12 accumulated metabolites which constantly decreased from the distal to the proximal section of *Nanorana pleskei* in the negative and positive ion modes. The metabolite names are listed on the top of the subgraph. Several metabolite names are too long to display clearly, thus IDs which can be found in Supplementary Table S4 are used instead. tip (T), middle (M) and root (R).

Supplementary Figure S7. Expression boxplot of 20 accumulated metabolites which constantly increased from the distal to the proximal section of *Nanorana pleskei* in the negative ion mode. The metabolite names are listed on the top of the subgraph. Several metabolite names are too long to display clearly, thus IDs which can be found in Supplementary Table S4 are used instead. tip (T), middle (M) and root (R).

Supplementary Figure S8. Expression boxplot of 13 accumulated metabolites which constantly increased from the distal to the proximal section of *Nanorana pleskei* in the positive ion mode, expect the last subgraph displayed inverted V-type curve changes. The metabolite names are listed on the top of the subgraph. Several metabolite names are too long to display clearly, thus IDs which can be found in Supplementary Table S4 are used instead. tip (T), middle (M) and root (R).

Supplementary Figure S9. The boxplot of integrin alpha V (ITGAV, K06487), integrin beta 3 (ITGB3, K06493) and mitogen-activated protein kinase 1/3 (MAPK1/3, K04371).

Supplementary Figure S10. The metabolites strongly related to integrin alpha V (ITGAV, K06487), integrin beta 3 (ITGB3, K06493) and mitogen-activated protein kinase 1/3 (MAPK1/3, K04371). The value of correlations ≥ 0.8.

Supplementary Figure S11. The metabolites strongly related to integrin alpha V (ITGAV, K06487), integrin beta 3 (ITGB3, K06493) and mitogen-activated protein kinase 1/3 (MAPK1/3, K04371). The value of correlations ≤ -0.8.

Supplementary Figure S12. The proteins related to the murder and suicide models.

Supplementary Figure S13. Correlation networks between the proteins of proteasome (ko03050) and metabolites with the absolute value of correlations ≥ 0.8.

Supplementary Figure S14. The 28 metabolites in the integrative analysis of protein and metabolite linkage (spearman correlation). The absolute values of the correlation coefficient of proteins and metabolites were above 0.9.

Supplementary Figure S15. The positively correlated proteins in the integrative analysis of protein and metabolite linkage (spearman correlation). The absolute values of the correlation coefficient of proteins and metabolites were above 0.9.

Supplementary Figure S16. The negatively correlated proteins in the integrative analysis of protein and metabolite linkage (spearman correlation). The absolute values of the correlation coefficient of proteins and metabolites were above 0.9.

Supplementary Figure S17. A total of twenty proteins in the endocytosis (ko04144) and regulation of actin cytoskeleton (ko04810). Which were negatively correlated to the 28 metabolites in Supplementary Figure S14.

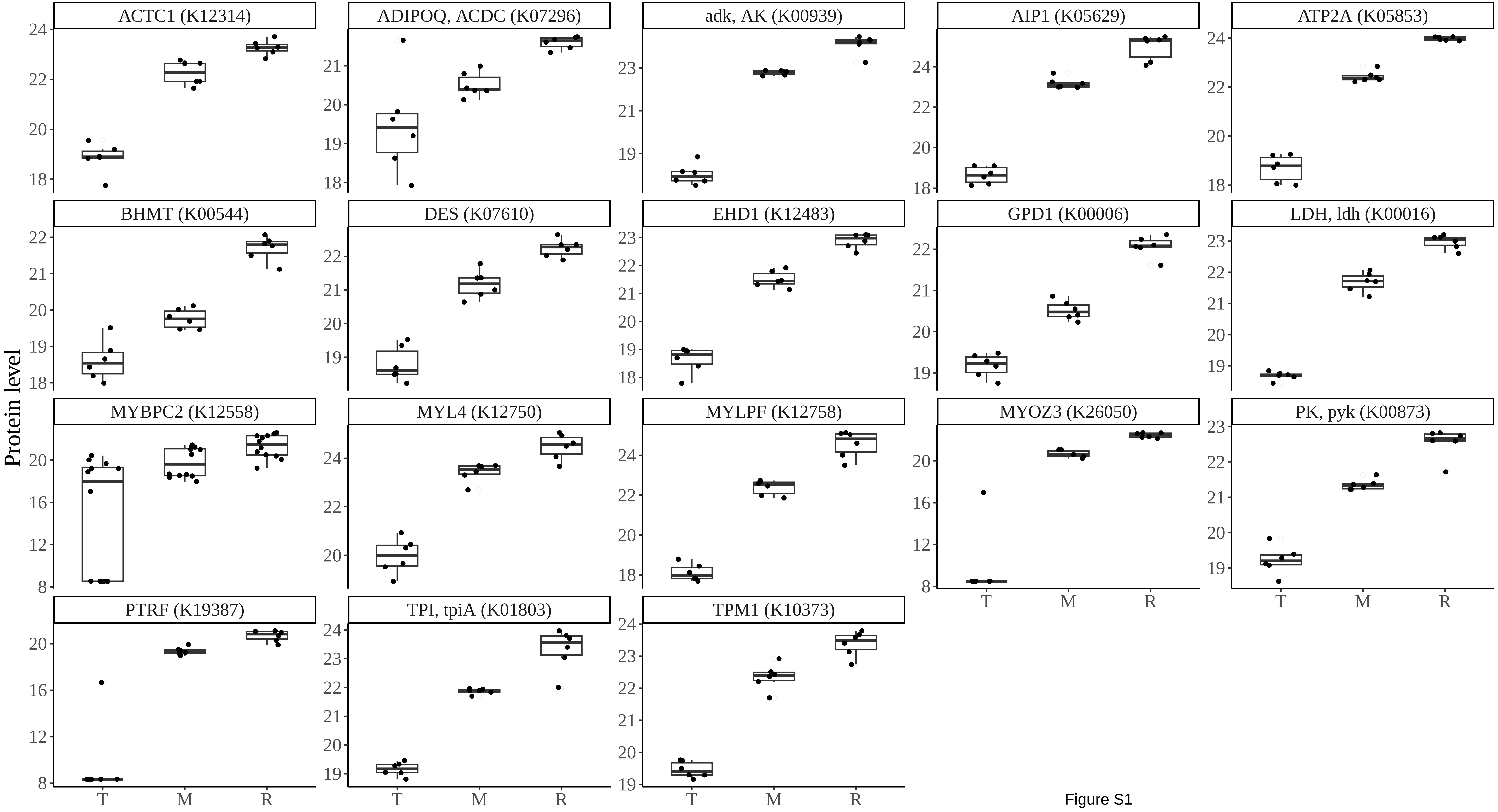
ACTC1 (K12314)
ADIPOQ, ACDC (K07296)
adk, AK (K00939)
AIP1 (K05629)
ATP2A (K05853)
BHMT (K00544)
DES (K07610)
EHD1 (K12483)
GPD1 (K00006)
LDH, ldh (K00016)
MYBPC2 (K12558)
MYL4 (K12750)
MYLPF (K12758)
MYOZ3 (K26050)
PK, pyk (K00873)
PTRF (K19387)
TPI, tpiA (K01803)
TPM1 (K10373)
Protein level
T
M
R

Figure S1

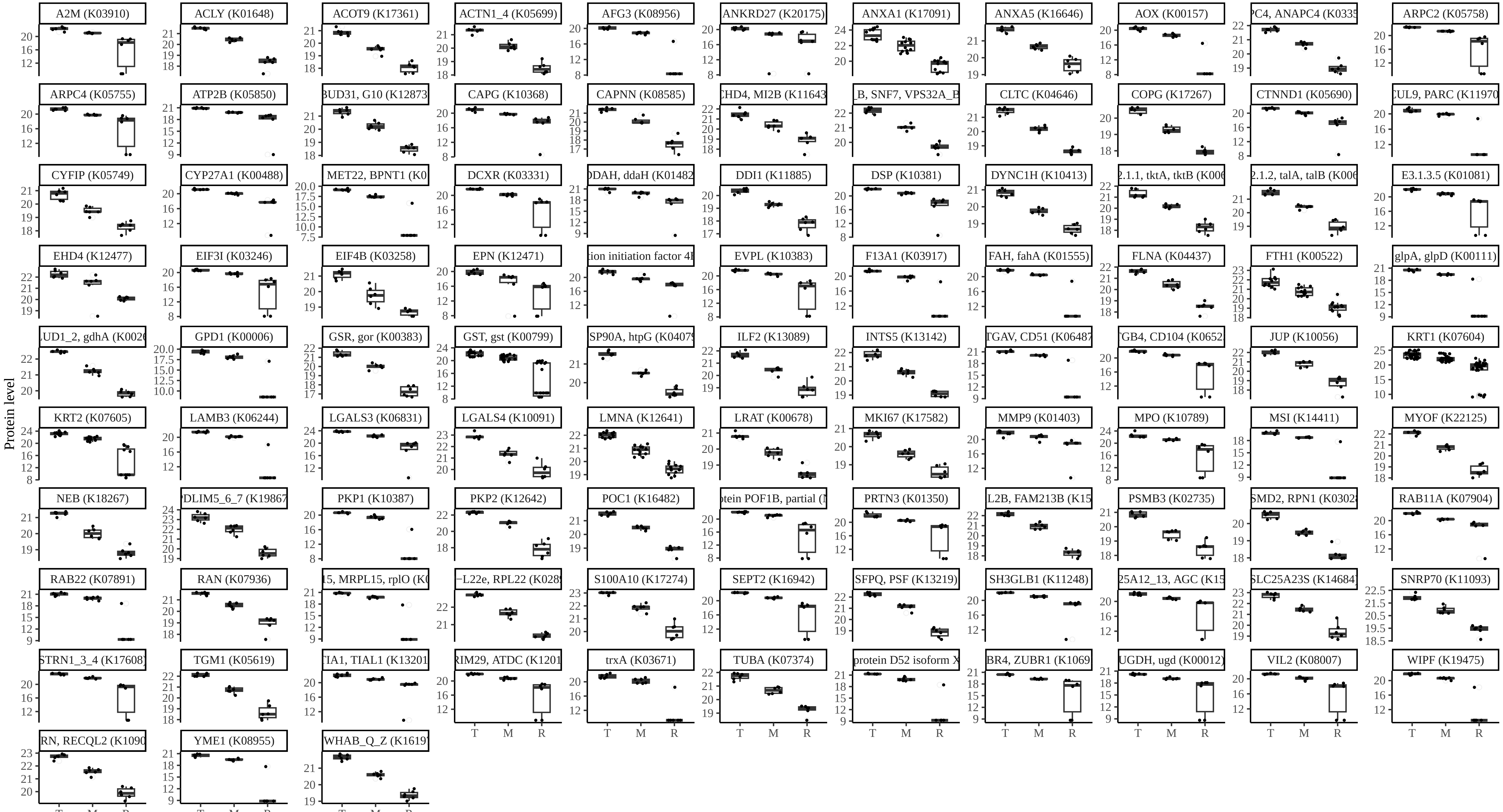
A2M (K03910)
ACLY (K01648)
ACOT9 (K17361)
ACTN1_4 (K05699)
AFG3 (K08956)
ANKRD27 (K20175)
ANXA1 (K17091)
ANXA5 (K16646)
AOX (K00157)
PC4, ANAPC4 (K0335
ARPC2 (K05758)
ARPC4 (K05755)
ATP2B (K05850)
BUD31, G10 (K12873
CAPG (K10368)
CAPNN (K08585)
CHD4, MI2B (K11643
_B, SNF7, VPS32A_B
CLTC (K04646)
COPG (K17267)
CTNND1 (K05690)
CUL9, PARC (K11970
CYFIP (K05749)
CYP27A1 (K00488)
MET22, BPNT1 (K0
DCXR (K03331)
DDAH, ddaH (K01482
DDI1 (K11885)
DSP (K10381)
DYNC1H (K10413)
2.1.1, tktA, tktB (K00
2.1.2, talA, talB (K00
E3.1.3.5 (K01081)
EHD4 (K12477)
EIF3I (K03246)
EIF4B (K03258)
EPN (K12471)
tion initiation factor 4
EVPL (K10383)
F13A1 (K03917)
FAH, fahA (K01555)
FLNA (K04437)
FTH1 (K00522)
glpA, glpD (K00111)
LUD1_2, gdhA (K002
GPD1 (K00006)
GSR, gor (K00383)
GST, gst (K00799)
SP90A, htpG (K0407
ILF2 (K13089)
INTS5 (K13142)
TGAV, CD51 (K06487
TGB4, CD104 (K0652
JUP (K10056)
KRT1 (K07604)
KRT2 (K07605)
LAMB3 (K06244)
LGALS3 (K06831)
LGALS4 (K10091)
LMNA (K12641)
LRAT (K00678)
MKI67 (K17582)
MMP9 (K01403)
MPO (K10789)
MSI (K14411)
MYOF (K22125)
NEB (K18267)
PDLIM5_6_7 (K19867
PKP1 (K10387)
PKP2 (K12642)
POC1 (K16482)
otein POF1B, partial (
PRTN3 (K01350)
L2B, FAM213B (K15
PSMB3 (K02735)
SMD2, RPN1 (K0302
RAB11A (K07904)
RAB22 (K07891)
RAN (K07936)
15, MRPL15, rplO (K
L22e, RPL22 (K028
S100A10 (K17274)
SEPT2 (K16942)
SFPQ, PSF (K13219)
SH3GLB1 (K11248)
25A12_13, AGC (K15
SLC25A23S (K14684)
SNRP70 (K11093)
STRN1_3_4 (K17608)
TGM1 (K05619)
TIA1, TIAL1 (K13201
RIM29, ATDC (K120
trxA (K03671)
TUBA (K07374)
protein D52 isoform X
BR4, ZUBR1 (K1069
UGDH, ugd (K00012)
VIL2 (K08007)
WIPF (K19475)
RN, RECQL2 (K1090
YME1 (K08955)
WHAB_Q_Z (K1619
Protein level
T
M
R

Figure S2

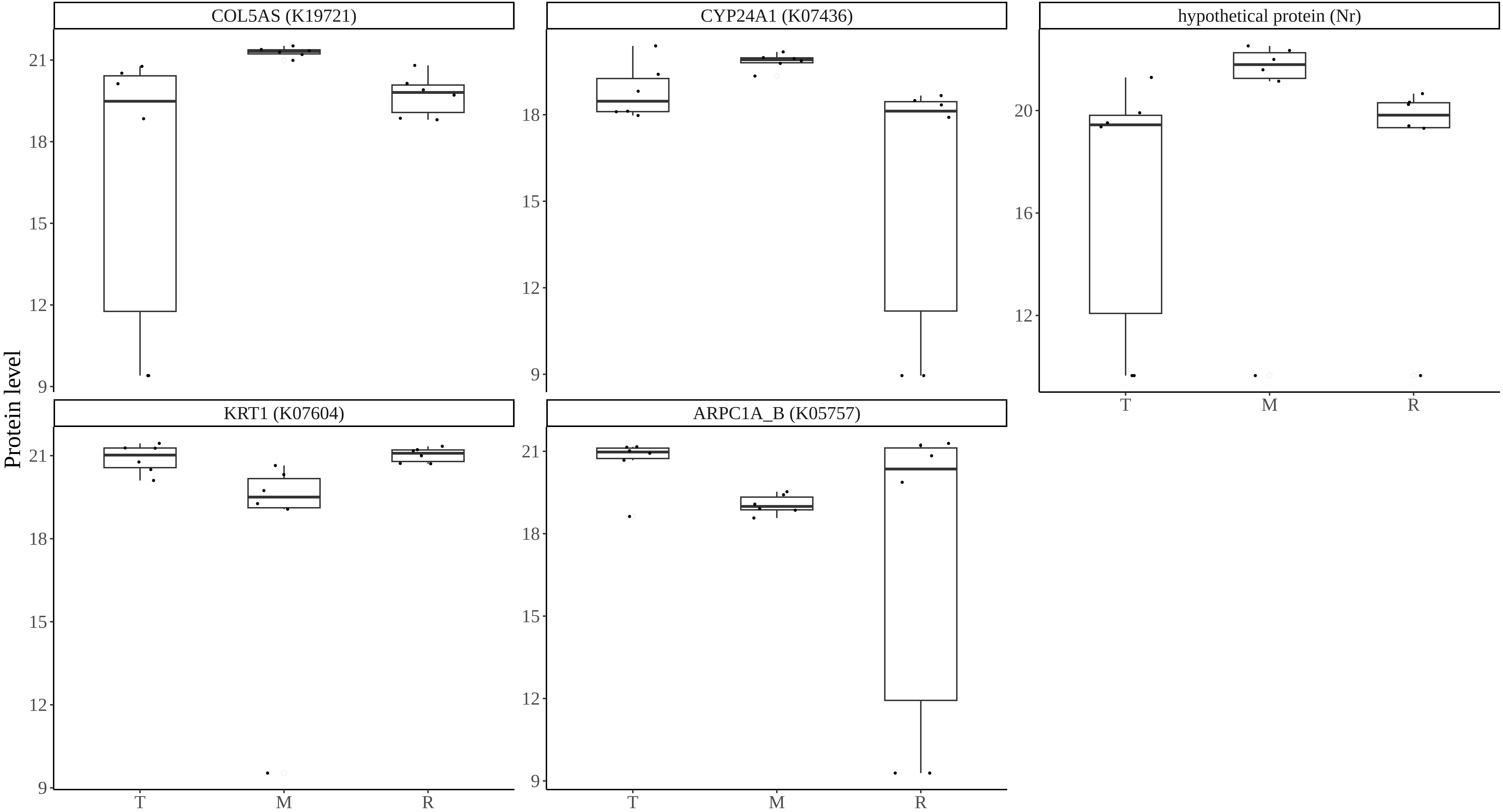
COL5AS (K19721)
CYP24A1 (K07436)
hypothetical protein (Nr)
KRT1 (K07604)
ARPC1A_B (K05757)
Protein level
T
M
R
21
18
15
12
9
20
16
12

Figure S3

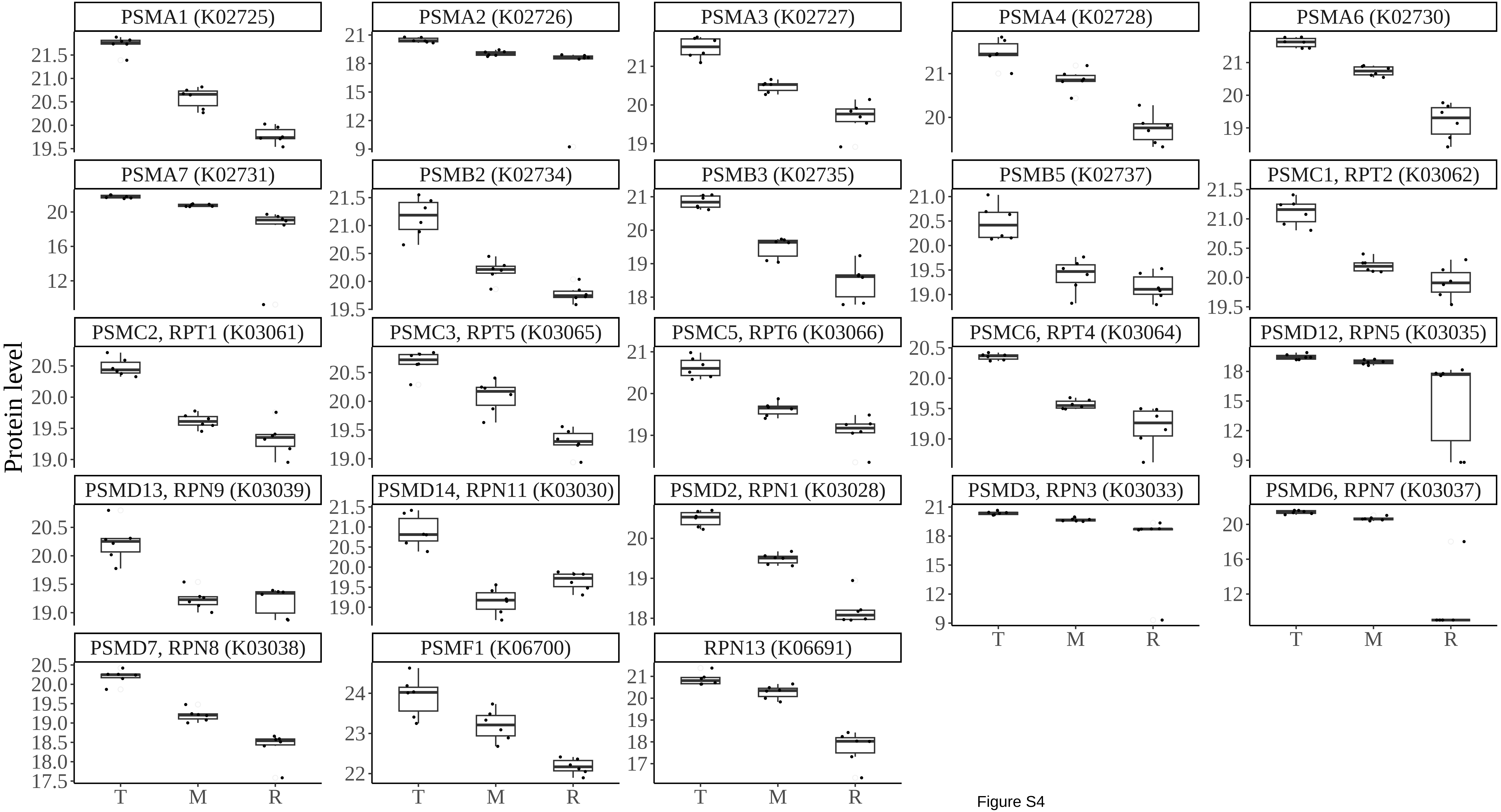
Protein level
PSMA1 (K02725)
PSMA2 (K02726)
PSMA3 (K02727)
PSMA4 (K02728)
PSMA6 (K02730)
PSMA7 (K02731)
PSMB2 (K02734)
PSMB3 (K02735)
PSMB5 (K02737)
PSMC1, RPT2 (K03062)
PSMC2, RPT1 (K03061)
PSMC3, RPT5 (K03065)
PSMC5, RPT6 (K03066)
PSMC6, RPT4 (K03064)
PSMD12, RPN5 (K03035)
PSMD13, RPN9 (K03039)
PSMD14, RPN11 (K03030)
PSMD2, RPN1 (K03028)
PSMD3, RPN3 (K03033)
PSMD6, RPN7 (K03037)
PSMD7, RPN8 (K03038)
PSMF1 (K06700)
RPN13 (K06691)
T
M
R

Figure S4

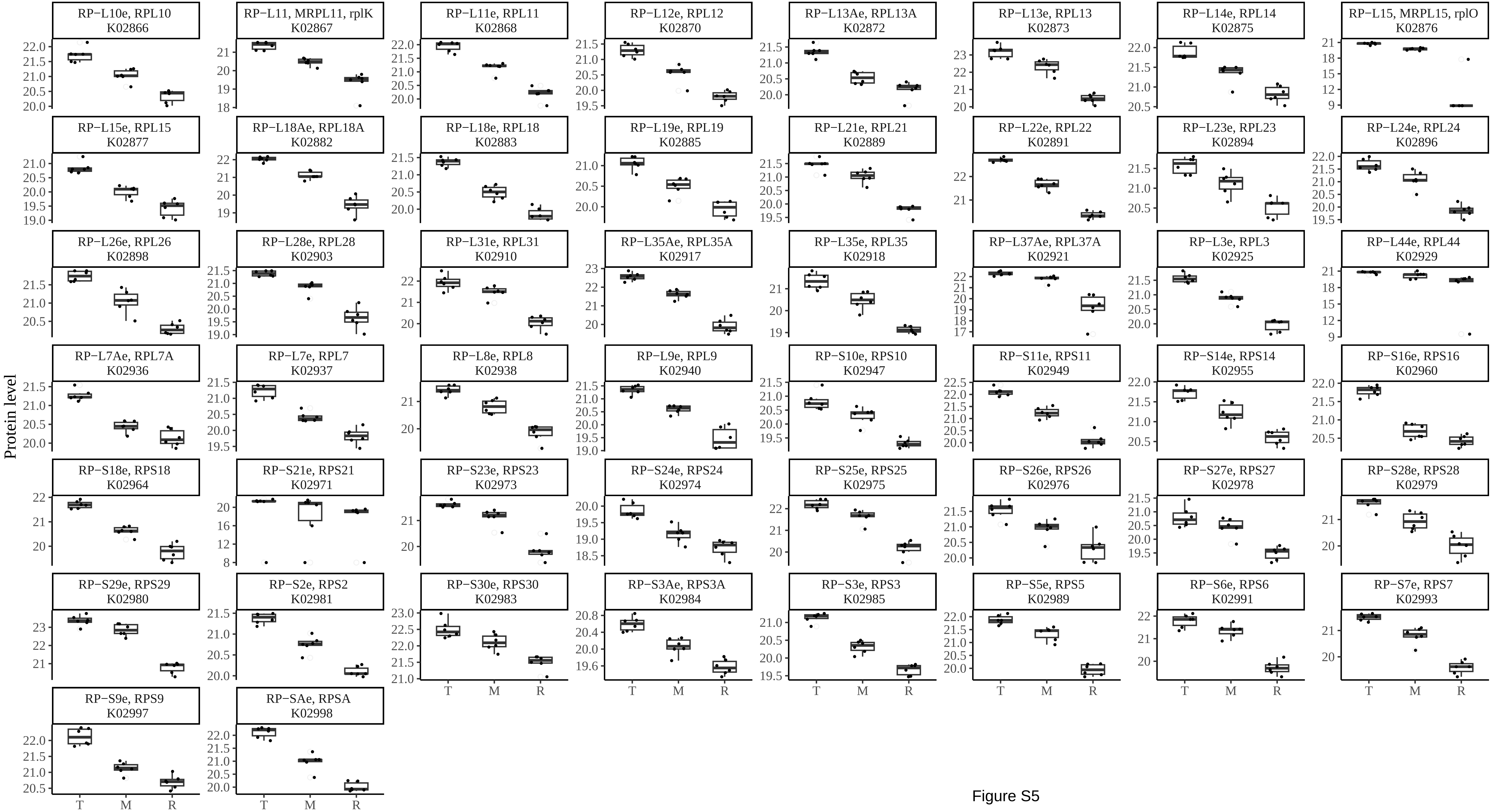
Protein level
RP−L10e, RPL10 K02866
RP−L11, MRPL11, rplK K02867
RP−L11e, RPL11 K02868
RP−L12e, RPL12 K02870
RP−L13Ae, RPL13A K02872
RP−L13e, RPL13 K02873
RP−L14e, RPL14 K02875
RP−L15, MRPL15, rplO K02876
RP−L15e, RPL15 K02877
RP−L18Ae, RPL18A K02882
RP−L18e, RPL18 K02883
RP−L19e, RPL19 K02885
RP−L21e, RPL21 K02889
RP−L22e, RPL22 K02891
RP−L23e, RPL23 K02894
RP−L24e, RPL24 K02896
RP−L26e, RPL26 K02898
RP−L28e, RPL28 K02903
RP−L31e, RPL31 K02910
RP−L35Ae, RPL35A K02917
RP−L35e, RPL35 K02918
RP−L37Ae, RPL37A K02921
RP−L3e, RPL3 K02925
RP−L44e, RPL44 K02929
RP−L7Ae, RPL7A K02936
RP−L7e, RPL7 K02937
RP−L8e, RPL8 K02938
RP−L9e, RPL9 K02940
RP−S10e, RPS10 K02947
RP−S11e, RPS11 K02949
RP−S14e, RPS14 K02955
RP−S16e, RPS16 K02960
RP−S18e, RPS18 K02964
RP−S21e, RPS21 K02971
RP−S23e, RPS23 K02973
RP−S24e, RPS24 K02974
RP−S25e, RPS25 K02975
RP−S26e, RPS26 K02976
RP−S27e, RPS27 K02978
RP−S28e, RPS28 K02979
RP−S29e, RPS29 K02980
RP−S2e, RPS2 K02981
RP−S30e, RPS30 K02983
RP−S3Ae, RPS3A K02984
RP−S3e, RPS3 K02985
RP−S5e, RPS5 K02989
RP−S6e, RPS6 K02991
RP−S7e, RPS7 K02993
RP−S9e, RPS9 K02997
RP−SAe, RPSA K02998
T
M
R

Figure S5

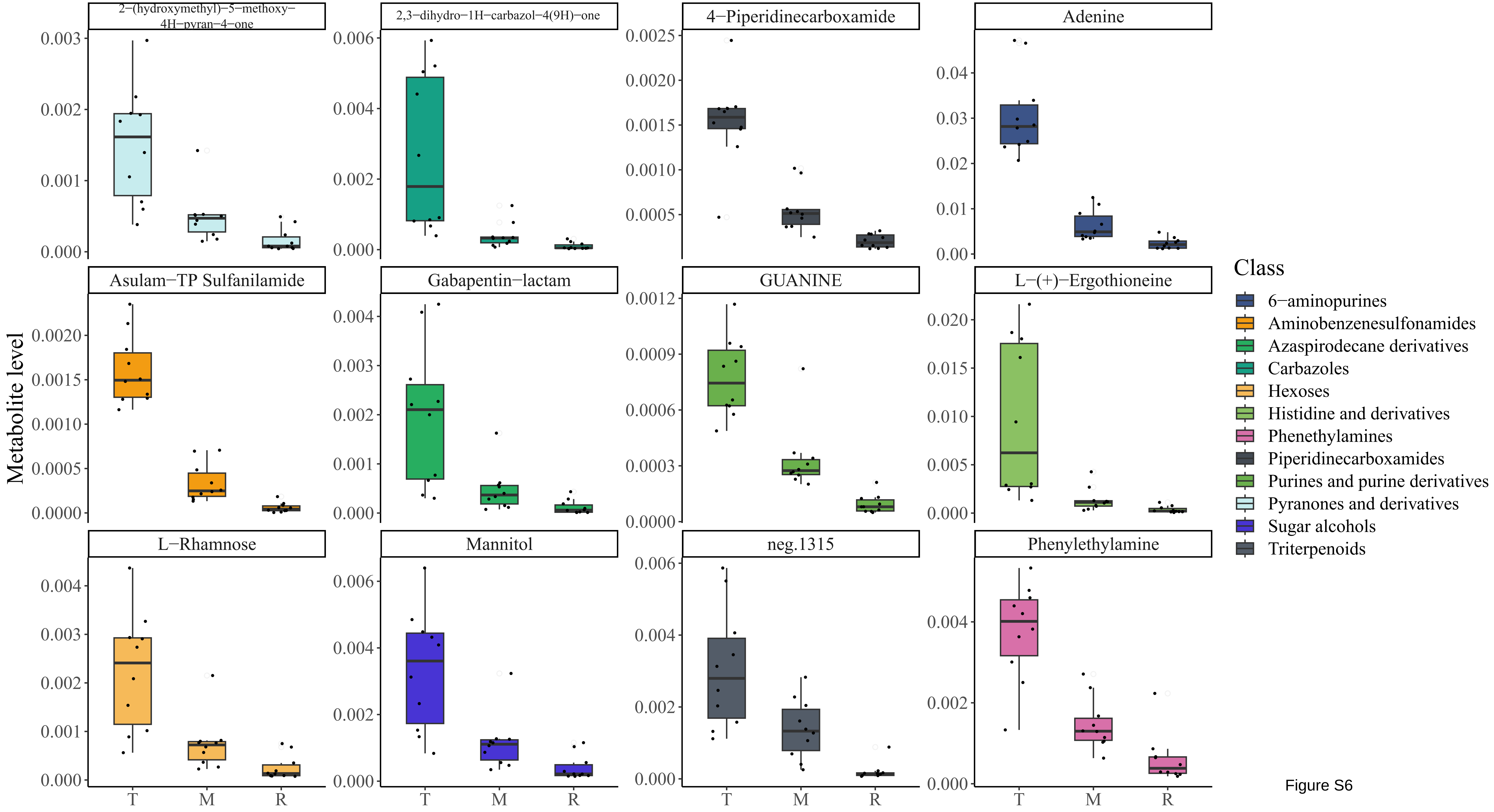
2−(hydroxymethyl)−5−methoxy−4H−pyran−4−one
2,3−dihydro−1H−carbazol−4(9H)−one
4−Piperidinecarboxamide
Adenine
Asulam−TP Sulfanilamide
Gabapentin−lactam
GUANINE
L−(+)−Ergothioneine
L−Rhamnose
Mannitol
neg.1315
Phenylethylamine
Metabolite level
T
M
R
Class
6−aminopurines
Aminobenzenesulfonamides
Azaspirodecane derivatives
Carbazoles
Hexoses
Histidine and derivatives
Phenethylamines
Piperidinecarboxamides
Purines and purine derivatives
Pyranones and derivatives
Sugar alcohols
Triterpenoids

Figure S6

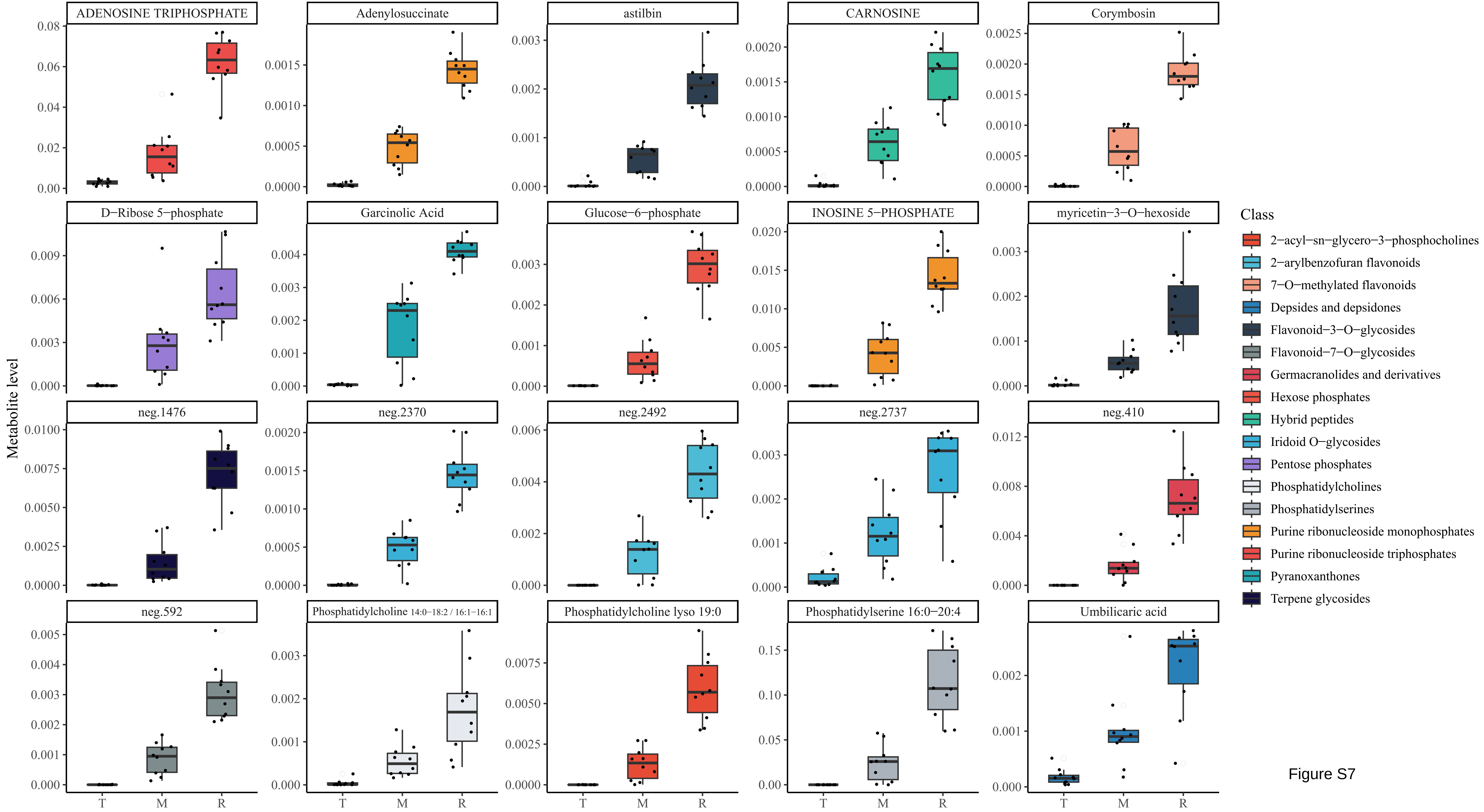
ADENOSINE TRIPHOSPHATE
Adenylosuccinate
astilbin
CARNOSINE
Corymbosin
D−Ribose 5−phosphate
Garcinolic Acid
Glucose−6−phosphate
INOSINE 5−PHOSPHATE
myricetin−3−O−hexoside
neg.1476
neg.2370
neg.2492
neg.2737
neg.410
neg.592
Phosphatidylcholine 14:0−18:2 / 16:1−16:1
Phosphatidylcholine lyso 19:0
Phosphatidylserine 16:0−20:4
Umbilicaric acid
Metabolite level
T
M
R
Class
2−acyl−sn−glycero−3−phosphocholines
2−arylbenzofuran flavonoids
7−O−methylated flavonoids
Depsides and depsidones
Flavonoid−3−O−glycosides
Flavonoid−7−O−glycosides
Germacranolides and derivatives
Hexose phosphates
Hybrid peptides
Iridoid O−glycosides
Pentose phosphates
Phosphatidylcholines
Phosphatidylserines
Purine ribonucleoside monophosphates
Purine ribonucleoside triphosphates
Pyranoxanthones
Terpene glycosides

Figure S7

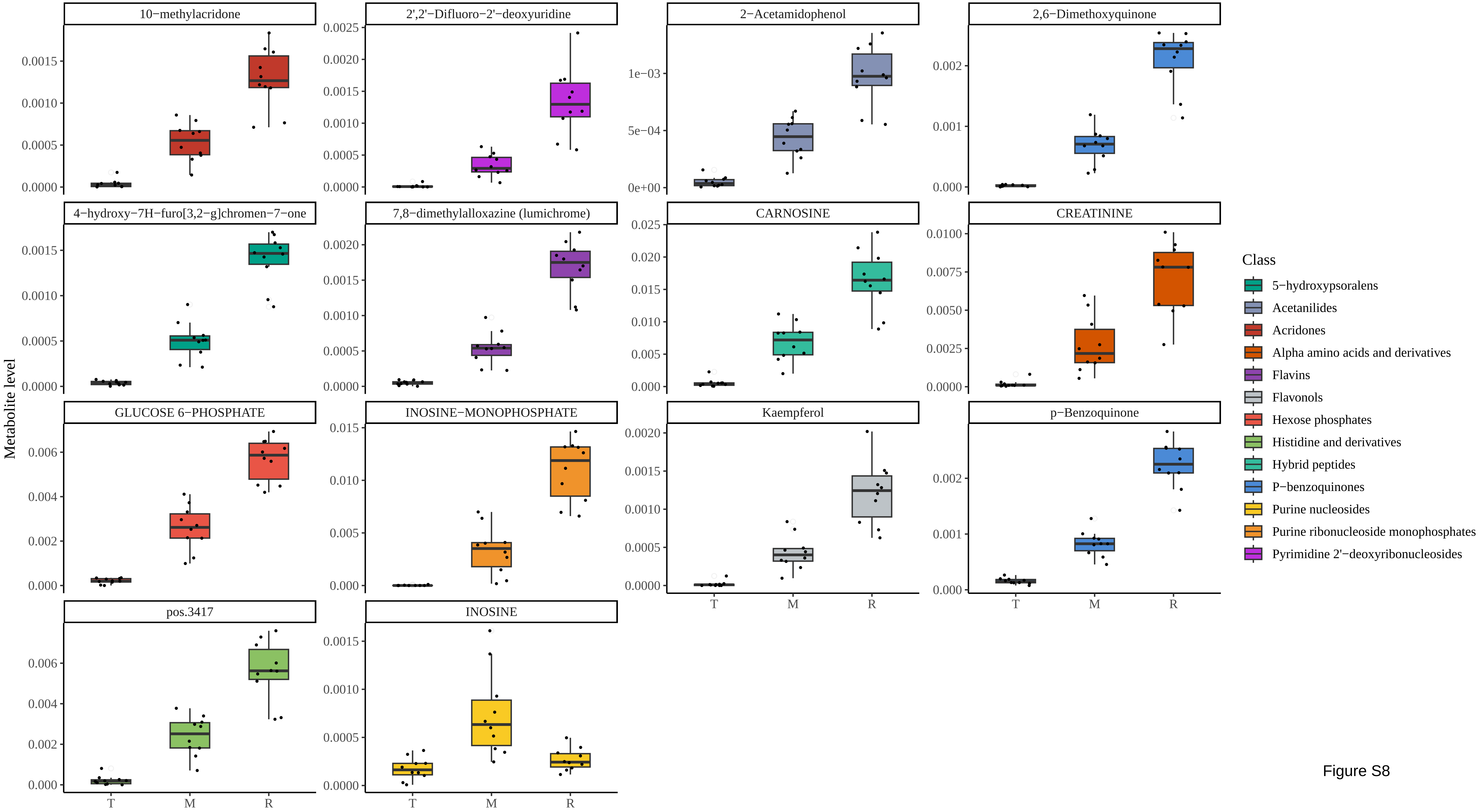
10−methylacridone
2',2'−Difluoro−2'−deoxyuridine
2−Acetamidophenol
2,6−Dimethoxyquinone
4−hydroxy−7H−furo[3,2−g]chromen−7−one
7,8−dimethylalloxazine (lumichrome)
CARNOSINE
CREATININE
GLUCOSE 6−PHOSPHATE
INOSINE−MONOPHOSPHATE
Kaempferol
p−Benzoquinone
pos.3417
INOSINE
Metabolite level
T
M
R
Class
5−hydroxypsoralens
Acetanilides
Acridones
Alpha amino acids and derivatives
Flavins
Flavonols
Hexose phosphates
Histidine and derivatives
Hybrid peptides
P−benzoquinones
Purine nucleosides
Purine ribonucleoside monophosphates
Pyrimidine 2'−deoxyribonucleosides

Figure S8

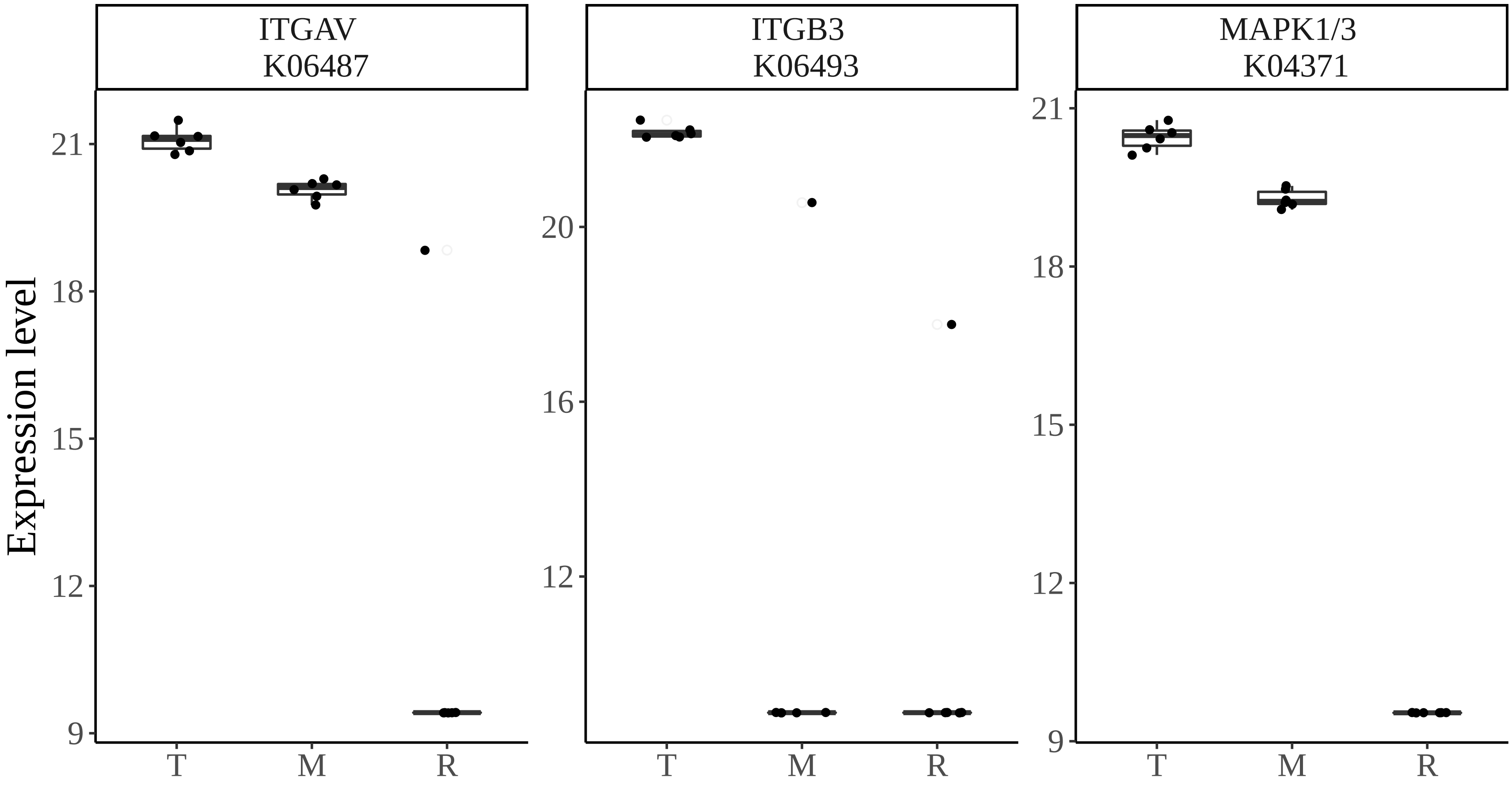
ITGAV
K06487
ITGB3
K06493
MAPK1/3
K04371
Expression level
21
18
15
12
9
20
16
12
21
18
15
12
9
T
M
R
T
M
R
T
M
R
Figure S9

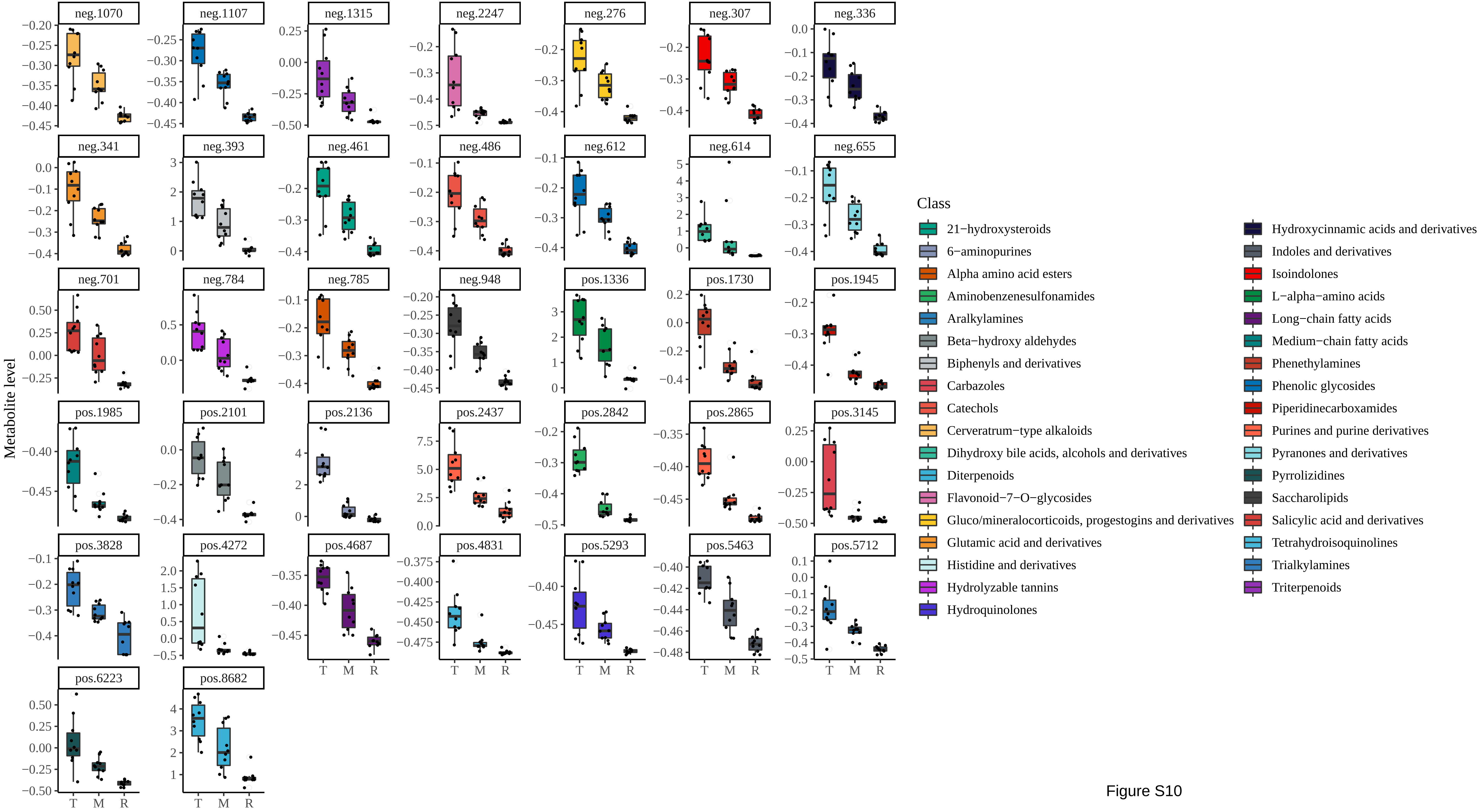
neg.1070
neg.1107
neg.1315
neg.2247
neg.276
neg.307
neg.336
neg.341
neg.393
neg.461
neg.486
neg.612
neg.614
neg.655
neg.701
neg.784
neg.785
neg.948
pos.1336
pos.1730
pos.1945
pos.1985
pos.2101
pos.2136
pos.2437
pos.2842
pos.2865
pos.3145
pos.3828
pos.4272
pos.4687
pos.4831
pos.5293
pos.5463
pos.5712
pos.6223
pos.8682
Metabolite level
T
M
R
Class
21−hydroxysteroids
6−aminopurines
Alpha amino acid esters
Aminobenzenesulfonamides
Aralkylamines
Beta−hydroxy aldehydes
Biphenyls and derivatives
Carbazoles
Catechols
Cerveratrum−type alkaloids
Dihydroxy bile acids, alcohols and derivatives
Diterpenoids
Flavonoid−7−O−glycosides
Gluco/mineralocorticoids, progestogins and derivatives
Glutamic acid and derivatives
Histidine and derivatives
Hydrolyzable tannins
Hydroquinolones
Hydroxycinnamic acids and derivatives
Indoles and derivatives
Isoindolones
L−alpha−amino acids
Long−chain fatty acids
Medium−chain fatty acids
Phenethylamines
Phenolic glycosides
Piperidinecarboxamides
Purines and purine derivatives
Pyranones and derivatives
Pyrrolizidines
Saccharolipids
Salicylic acid and derivatives
Tetrahydroisoquinolines
Trialkylamines
Triterpenoids

Figure S10

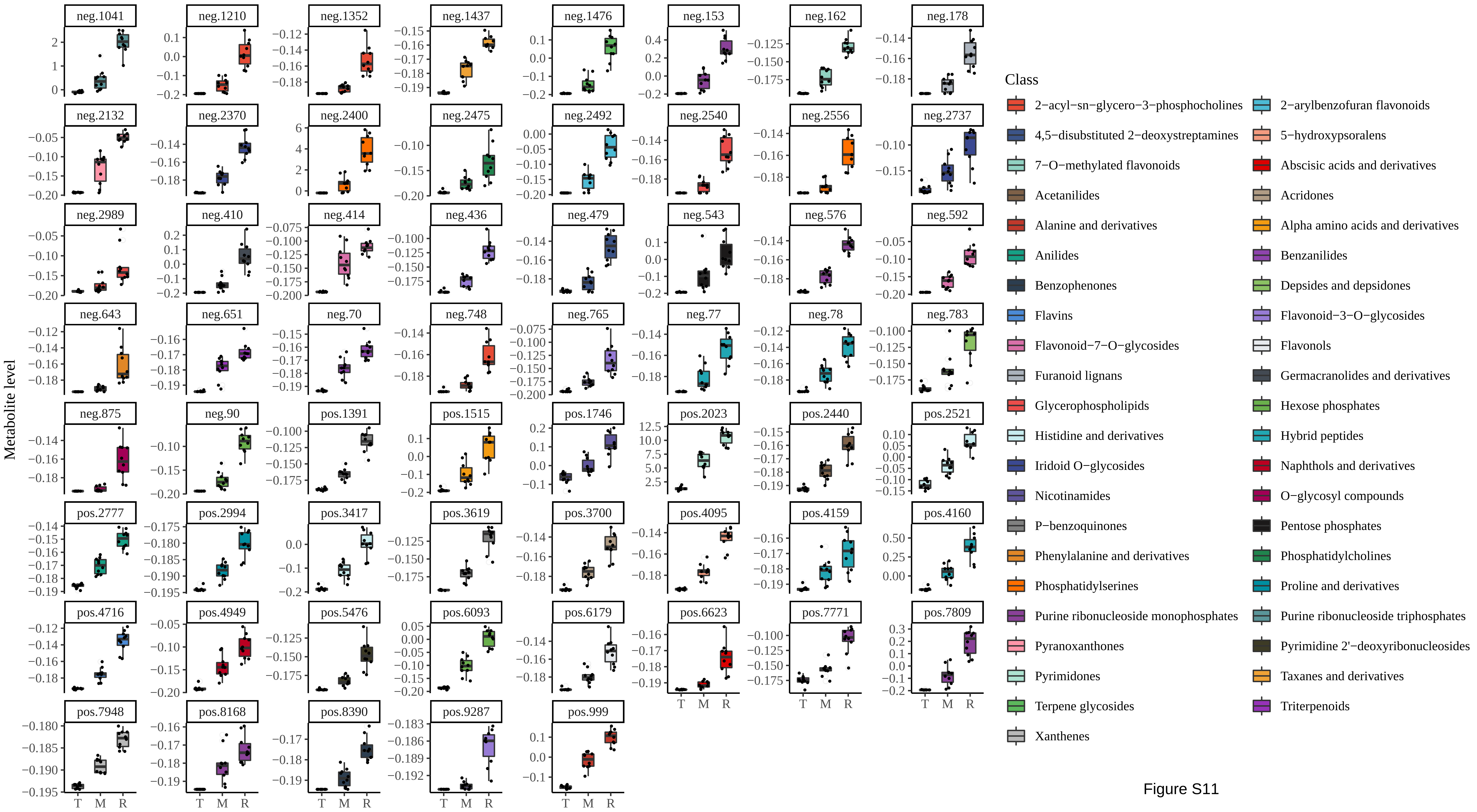
neg.1041
neg.1210
neg.1352
neg.1437
neg.1476
neg.153
neg.162
neg.178
neg.2132
neg.2370
neg.2400
neg.2475
neg.2492
neg.2540
neg.2556
neg.2737
neg.2989
neg.410
neg.414
neg.436
neg.479
neg.543
neg.576
neg.592
neg.643
neg.651
neg.70
neg.748
neg.765
neg.77
neg.78
neg.783
neg.875
neg.90
pos.1391
pos.1515
pos.1746
pos.2023
pos.2440
pos.2521
pos.2777
pos.2994
pos.3417
pos.3619
pos.3700
pos.4095
pos.4159
pos.4160
pos.4716
pos.4949
pos.5476
pos.6093
pos.6179
pos.6623
pos.7771
pos.7809
pos.7948
pos.8168
pos.8390
pos.9287
pos.999
Metabolite level
T
M
R
Class
2−acyl−sn−glycero−3−phosphocholines
2−arylbenzofuran flavonoids
4,5−disubstituted 2−deoxystreptamines
5−hydroxypsoralens
7−O−methylated flavonoids
Abscisic acids and derivatives
Acetanilides
Acridones
Alanine and derivatives
Alpha amino acids and derivatives
Anilides
Benzanilides
Benzophenones
Depsides and depsidones
Flavins
Flavonoid−3−O−glycosides
Flavonoid−7−O−glycosides
Flavonols
Furanoid lignans
Germacranolides and derivatives
Glycerophospholipids
Hexose phosphates
Histidine and derivatives
Hybrid peptides
Iridoid O−glycosides
Naphthols and derivatives
Nicotinamides
O−glycosyl compounds
P−benzoquinones
Pentose phosphates
Phenylalanine and derivatives
Phosphatidylcholines
Phosphatidylserines
Proline and derivatives
Purine ribonucleoside monophosphates
Purine ribonucleoside triphosphates
Pyranoxanthones
Pyrimidine 2'−deoxyribonucleosides
Pyrimidones
Taxanes and derivatives
Terpene glycosides
Triterpenoids
Xanthenes

Figure S11

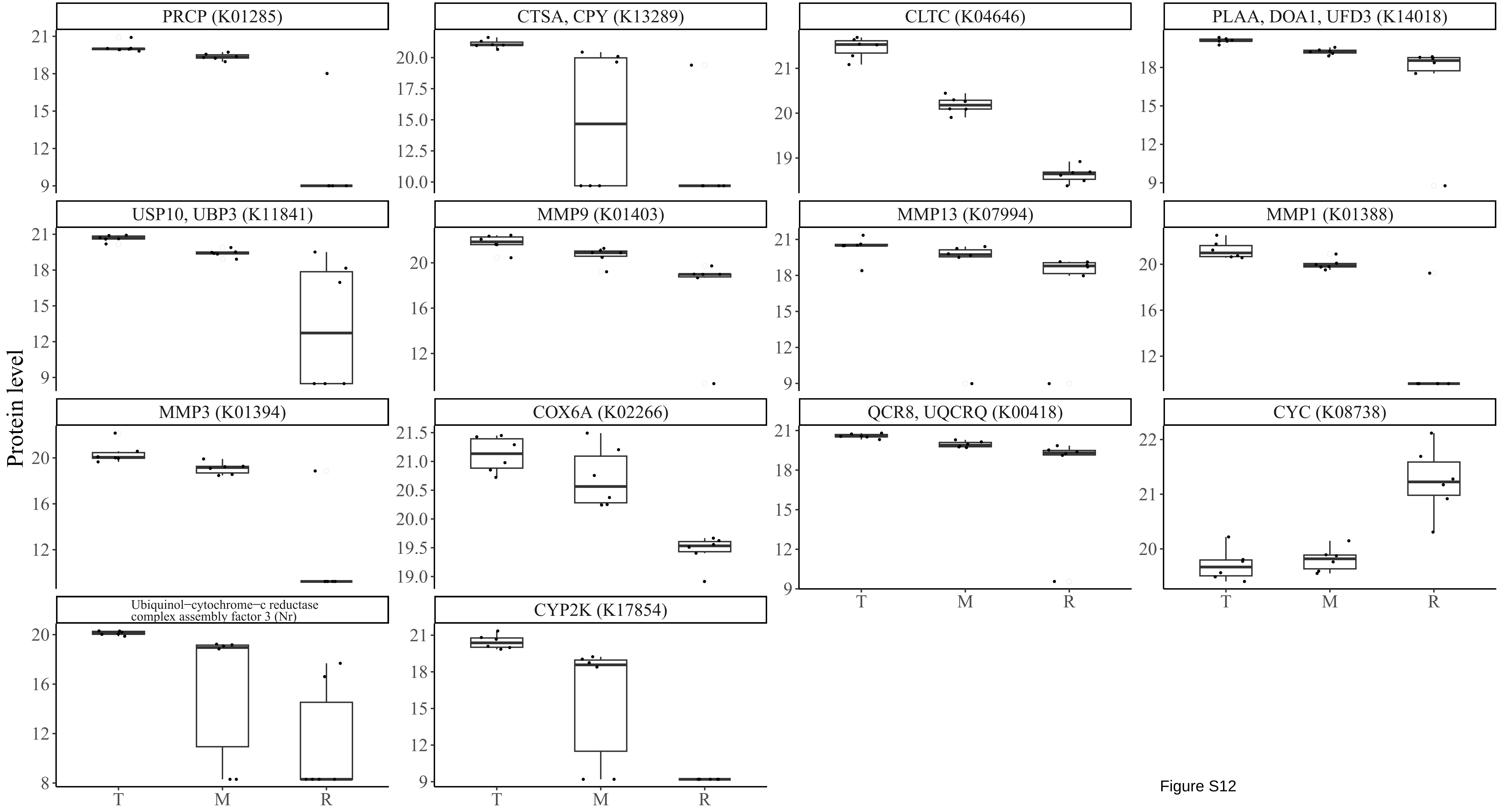
PRCP (K01285)
CTSA, CPY (K13289)
CLTC (K04646)
PLAA, DOA1, UFD3 (K14018)
USP10, UBP3 (K11841)
MMP9 (K01403)
MMP13 (K07994)
MMP1 (K01388)
MMP3 (K01394)
COX6A (K02266)
QCR8, UQCRQ (K00418)
CYC (K08738)
Ubiquinol−cytochrome−c reductase complex assembly factor 3 (Nr)
CYP2K (K17854)
Protein level
T
M
R

Figure S12

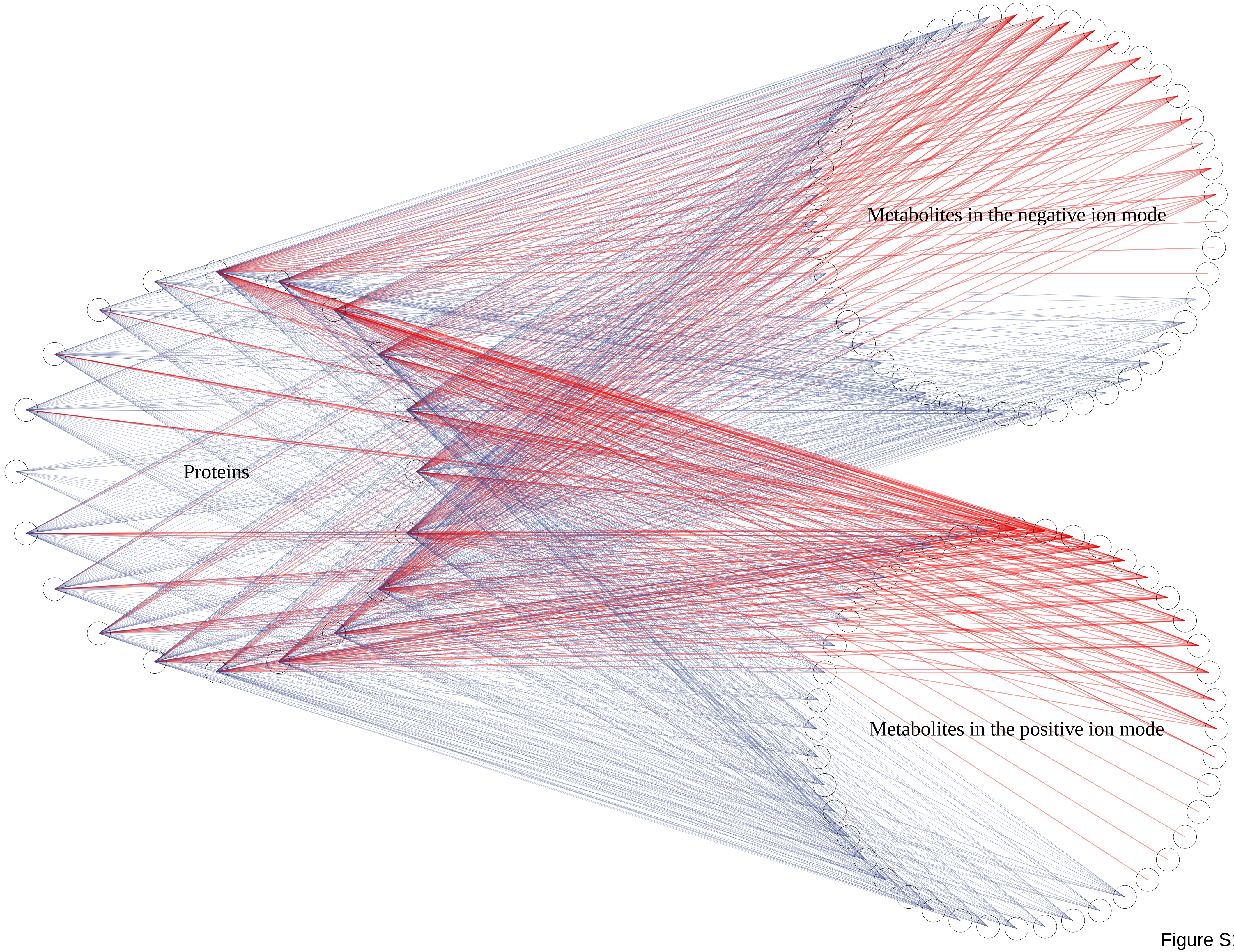
Metabolites in the negative ion mode
Proteins
Metabolites in the positive ion mode

Figure S13

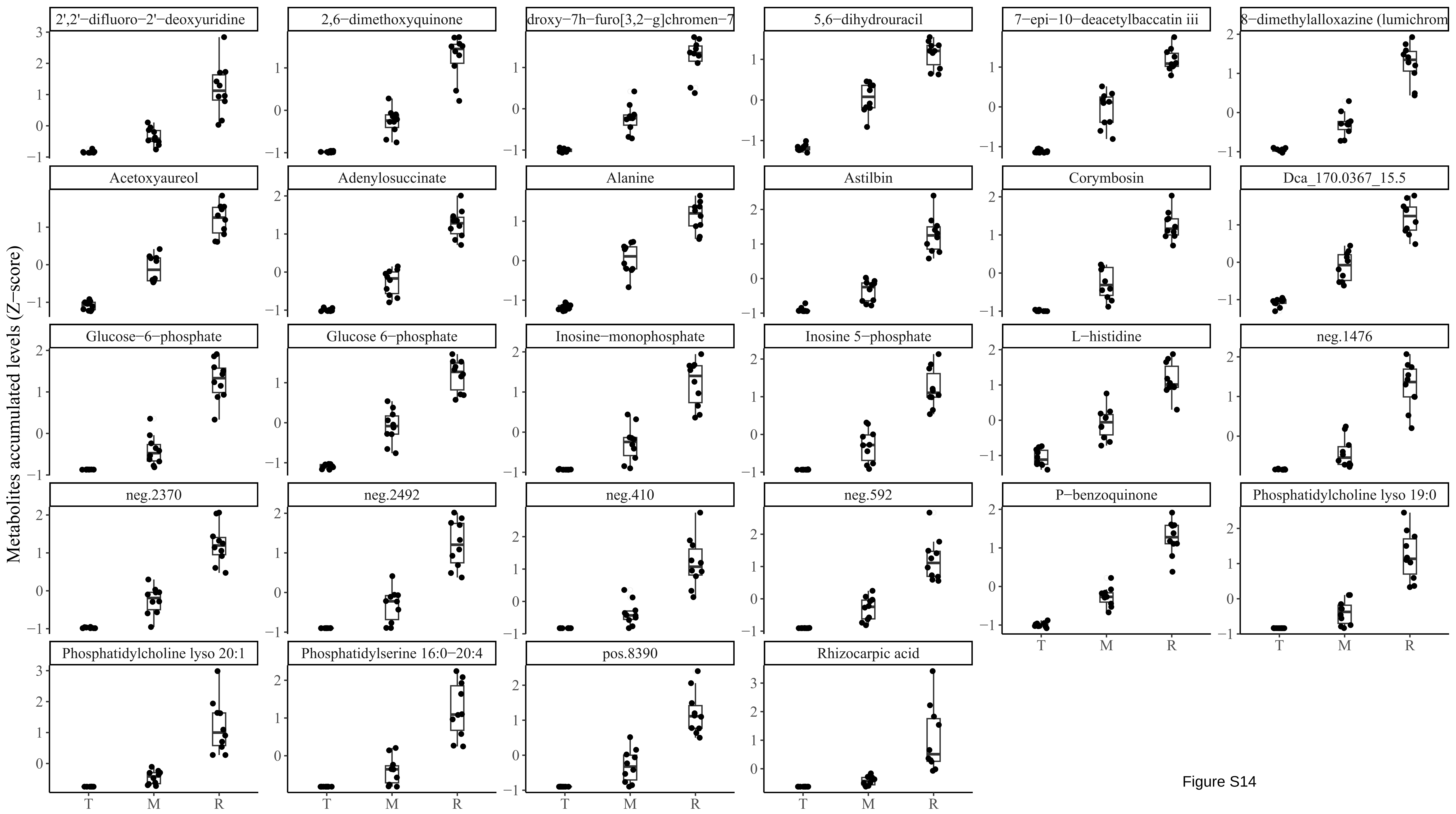

Metabolites accumulated levels (Z−score)
2',2'−difluoro−2'−deoxyuridine
2,6−dimethoxyquinone
droxy−7h−furo[3,2−g]chromen−7
5,6−dihydrouracil
7−epi−10−deacetylbaccatin iii
8−dimethylalloxazine (lumichrom
Acetoxyaureol
Adenylosuccinate
Alanine
Astilbin
Corymbosin
Dca_170.0367_15.5
Glucose−6−phosphate
Glucose 6−phosphate
Inosine−monophosphate
Inosine 5−phosphate
L−histidine
neg.1476
neg.2370
neg.2492
neg.410
neg.592
P−benzoquinone
Phosphatidylcholine lyso 19:0
Phosphatidylcholine lyso 20:1
Phosphatidylserine 16:0−20:4
pos.8390
Rhizocarpic acid
T
M
R

Figure S14

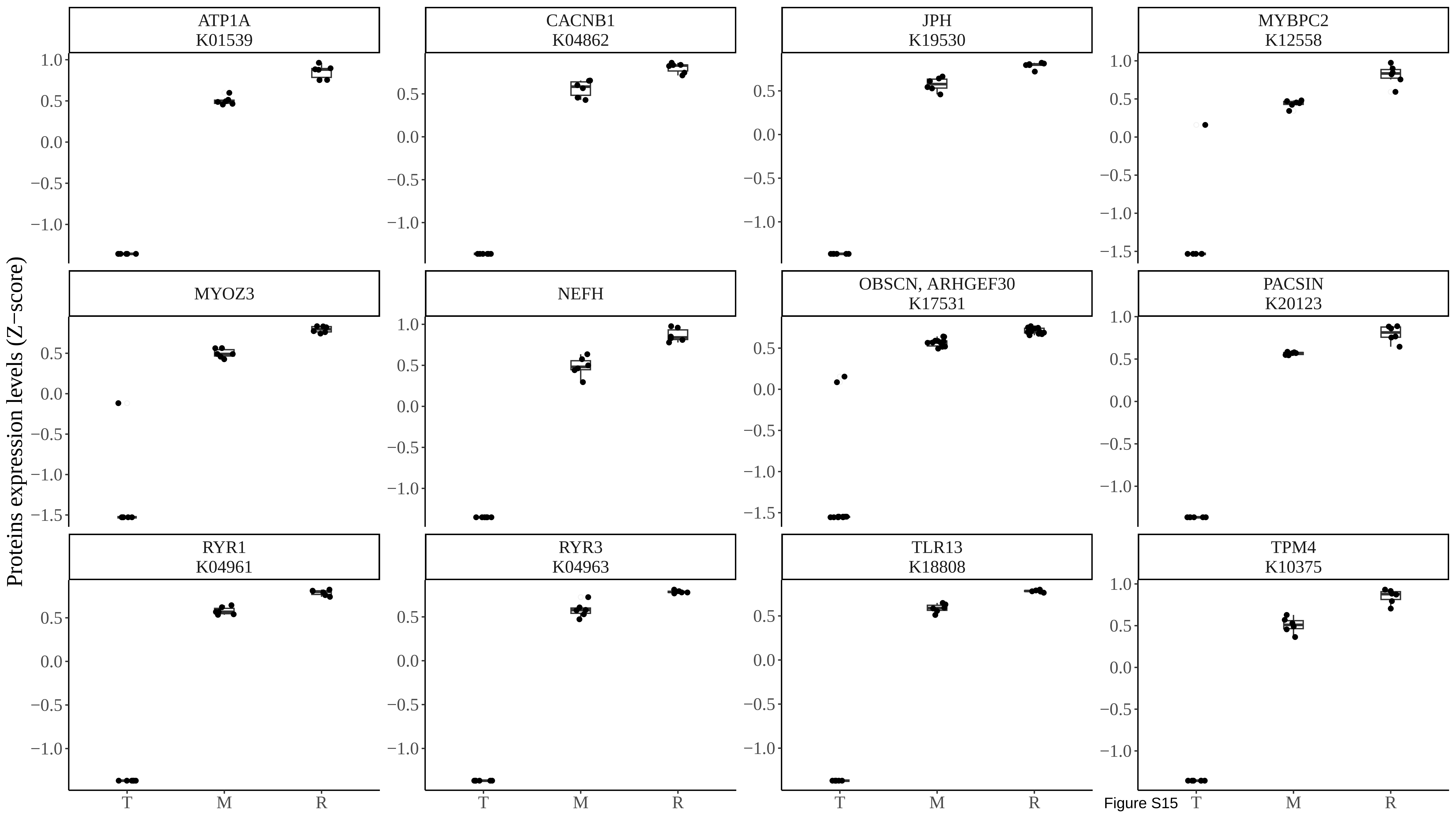
ATP1A
K01539
CACNB1
K04862
JPH
K19530
MYBPC2
K12558
MYOZ3
NEFH
OBSCN, ARHGEF30
K17531
PACSIN
K20123
RYR1
K04961
RYR3
K04963
TLR13
K18808
TPM4
K10375
Proteins expression levels (Z−score)
T
M
R

Figure S15

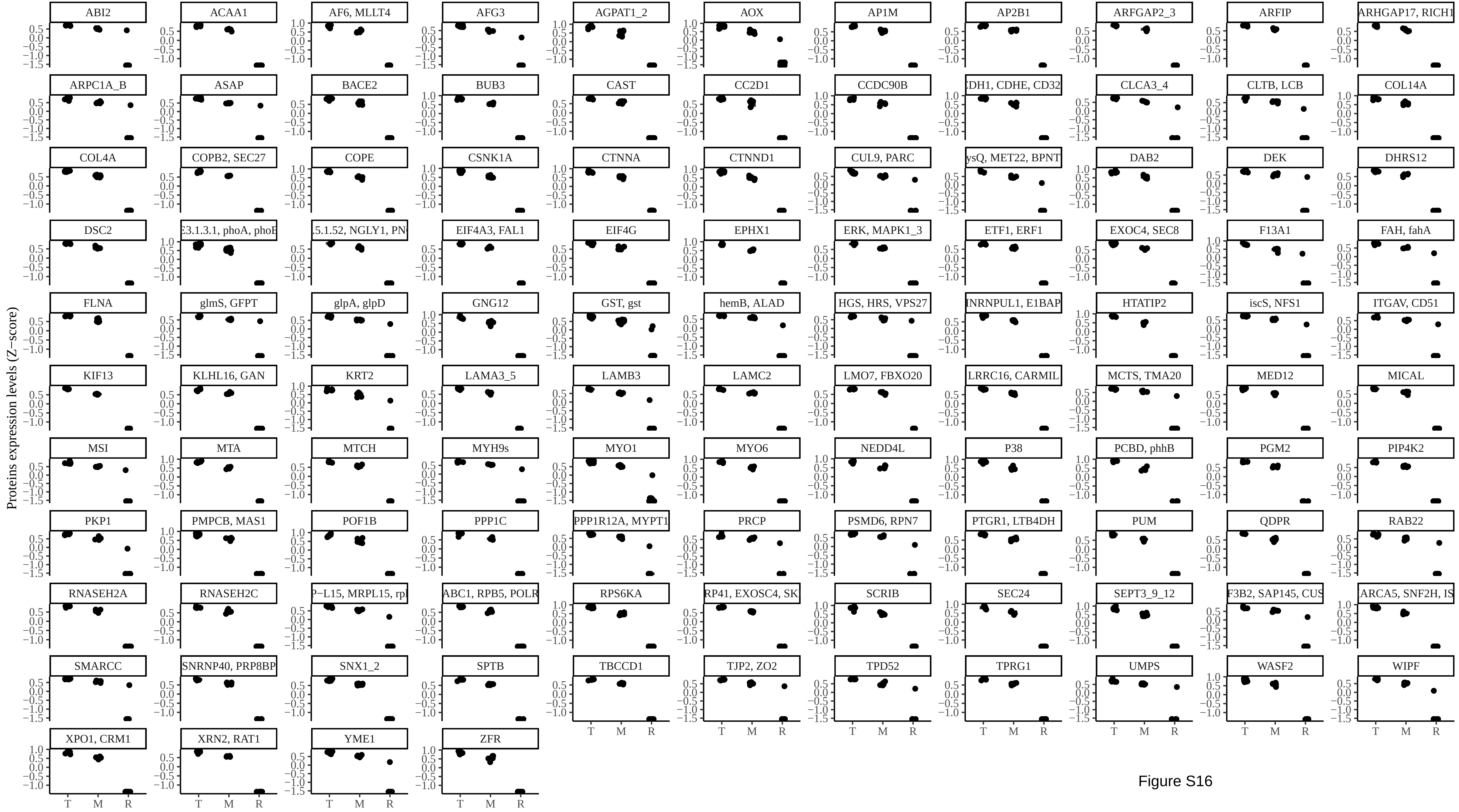
Proteins expression levels (Z−score)
ABI2
ACAA1
AF6, MLLT4
AFG3
AGPAT1_2
AOX
AP1M
AP2B1
ARFGAP2_3
ARFIP
ARHGAP17, RICH1
ARPC1A_B
ASAP
BACE2
BUB3
CAST
CC2D1
CCDC90B
CDH1, CDHE, CD32
CLCA3_4
CLTB, LCB
COL14A
COL4A
COPB2, SEC27
COPE
CSNK1A
CTNNA
CTNND1
CUL9, PARC
ysQ, MET22, BPNT
DAB2
DEK
DHRS12
DSC2
E3.1.3.1, phoA, phoE
.5.1.52, NGLY1, PN
EIF4A3, FAL1
EIF4G
EPHX1
ERK, MAPK1_3
ETF1, ERF1
EXOC4, SEC8
F13A1
FAH, fahA
FLNA
glmS, GFPT
glpA, glpD
GNG12
GST, gst
hemB, ALAD
HGS, HRS, VPS27
INRNPUL1, E1BAP
HTATIP2
iscS, NFS1
ITGAV, CD51
KIF13
KLHL16, GAN
KRT2
LAMA3_5
LAMB3
LAMC2
LMO7, FBXO20
LRRC16, CARMIL
MCTS, TMA20
MED12
MICAL
MSI
MTA
MTCH
MYH9s
MYO1
MYO6
NEDD4L
P38
PCBD, phhB
PGM2
PIP4K2
PKP1
PMPCB, MAS1
POF1B
PPP1C
PPP1R12A, MYPT1
PRCP
PSMD6, RPN7
PTGR1, LTB4DH
PUM
QDPR
RAB22
RNASEH2A
RNASEH2C
P−L15, MRPL15, rpl
ABC1, RPB5, POLR
RPS6KA
RP41, EXOSC4, SK
SCRIB
SEC24
SEPT3_9_12
F3B2, SAP145, CUS
ARCA5, SNF2H, IS
SMARCC
SNRNP40, PRP8BP
SNX1_2
SPTB
TBCCD1
TJP2, ZO2
TPD52
TPRG1
UMPS
WASF2
WIPF
XPO1, CRM1
XRN2, RAT1
YME1
ZFR
T
M
R

Figure S16

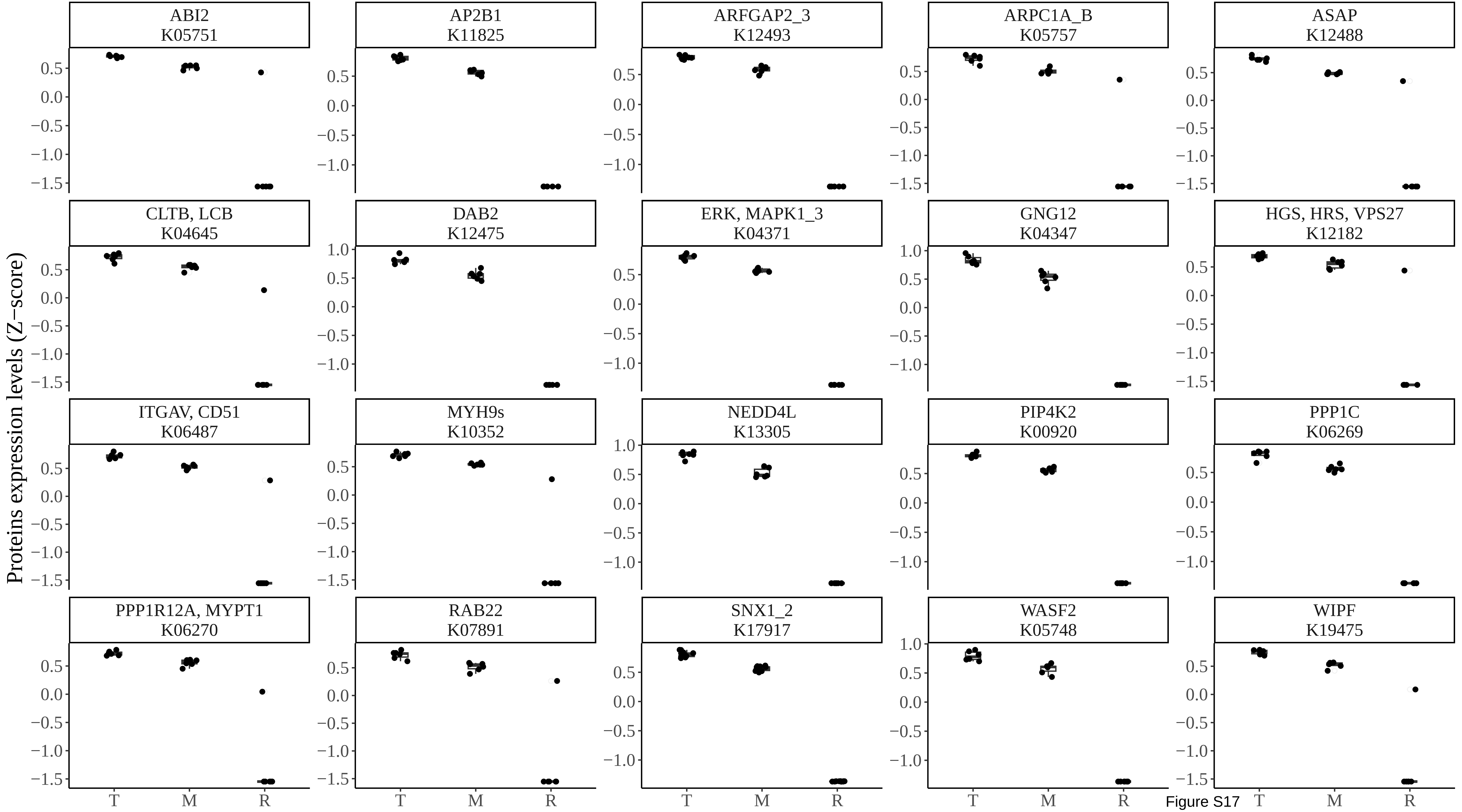
ABI2
K05751
AP2B1
K11825
ARFGAP2_3
K12493
ARPC1A_B
K05757
ASAP
K12488
CLTB, LCB
K04645
DAB2
K12475
ERK, MAPK1_3
K04371
GNG12
K04347
HGS, HRS, VPS27
K12182
ITGAV, CD51
K06487
MYH9s
K10352
NEDD4L
K13305
PIP4K2
K00920
PPP1C
K06269
PPP1R12A, MYPT1
K06270
RAB22
K07891
SNX1_2
K17917
WASF2
K05748
WIPF
K19475
Proteins expression levels (Z−score)
T
M
R

Figure S17
